# Reconceptualizing peptic ulcers as a psychosomatic disorder: a new etiological theory resolving a long-standing controversy surrounding *Helicobacter pylori*

**DOI:** 10.3389/fpsyt.2026.1749047

**Published:** 2026-05-15

**Authors:** Simon Xin Min Dong

**Affiliations:** Department of Research and Development, International Institute of Consciousness Science^®^, Vancouver, BC, Canada

**Keywords:** peptic ulcers, etiology, pathogenesis, *Helicobacter pylori*, Complex Causal Relationship, psychosomatic disease, psychological stress, three-phase psycho-neuropathological model

## Abstract

In the 1980s, Marshall proposed that peptic ulcers are an infectious disease caused by *Helicobacter pylori*. However, this etiology has remained controversial in the literature and faces persistent challenges: the bacterium’s ulcerogenic mechanism remains unknown, and eradication does not consistently prevent relapses, particularly in *Helicobacter pylori*-negative cases. Critically, this prevailing theory struggles to explain 15 major characteristics and 75 (92.6%) of 81 key observations/phenomena associated with the disease. This study synthesizes all the five major historical theories, including Marshall’s, into a more comprehensive etiological theory, *Theory of Nodes*, which hypothesizes that peptic ulcers are a psychosomatic disease triggered by psychological stress. Detailed in a six-article series, this emerging theory provides a comprehensive framework explaining the 15 characteristics and 81 observations/phenomena, addressing long-standing controversies and mysteries. Notably, its proposed interventions targeting psychosomatic and psychological mechanisms may effectively prevent disease onset and relapses. Employing a systematic approach, this sixth article aims to rigorously evaluate the two competing theories, resolving the four-decade-long controversy surrounding *Helicobacter pylori*’s role in peptic ulcers. Guided by a three-phase psycho-neuropathological model derived from *Theory of Nodes*, this comparative analysis suggests that all the three lines of evidence supporting *Helicobacter pylori* as a causative agent are inconclusive, and two of three other attributed observations may be subject to alternative interpretation. Moreover, historical definitions of “etiological factor” and “causality”, alongside epidemiological studies, disease characteristics, and prior observations, also dispute *Helicobacter pylori*’s causal role. Critically, the explanatory power of *Theory of Nodes* indicates that the bacterium’s contribution to ulceration is in fact a fortuitous secondary event, dependent on pre-existing mucosal lesions caused by a long-term psychosocial-neuropathological process. These findings suggest that the prevailing belief in *Helicobacter pylori*’s causal role in peptic ulcers might be a misconception, one that may have diverted ulcer research from more productive avenues for four decades. Recognizing and correcting such misconceptions is essential to overcoming barriers in medical research. Most importantly, this study uncovers the psychosomatic origins of various diseases, thereby paving the way for a new era of medicine focused on etiological understanding and prevention. Unequivocally, this may accelerate the progress of life science and medicine.

## 1 Introduction

The pathogenesis of peptic ulcers (including duodenal and gastric ulcers) has been a subject of intense debate for centuries, leading to 13 etiological theories in modern medical history, encompassing Schwartz’s *‘No acid, no ulcer*’ in 1910, Von Bergmann’s *Nerve Theory* in 1913, and the *Psychosomatic Theory* and *Stress Theory* introduced by Alexander and Selye in 1950 (1). Although each of them could explain a few of the 15 major characteristics and 81 key observations/phenomena of the disease, they all failed to address the majority due to their respective inconsistencies. To date, none has explained the birth-cohort phenomenon (2) and seasonal variation of peptic ulcers (3), along with morphology and predilection sites, perforation and bleeding, and the relapses and multiplicity of gastric ulcers. Moreover, although duodenal and gastric ulcers share much in common, they are epidemiologically, behaviorally, and genetically different (4, 5). The similarities and differences between duodenal and gastric ulcers remain elusive (6).

The isolation of *Helicobacter pylori* (*H. pylori*) in 1982 dramatically changed the concept of peptic ulcers (7, 8). In 1987, Marshall proposed that peptic ulcers are an infectious disease caused by *H. pylori* (9), and in 1988, he further concluded that ‘*H. pylori* is the most important etiological factor so far described for duodenal ulcer’ (10). This proposed causal relationship is primarily supported by three lines of evidence (10, 11). First, most ulcer patients are infected with *H. pylori*, and clinical patients have a higher infection rate than the general population (12–14). Second, ulcers are significantly larger in infected rats, with infection delaying healing that is resolved by elimination of the bacterium (15, 16). Third, elimination of *H. pylori* results in a significant reduction in ulcer relapse rate (10, 11, 17). This etiological theory has been designated as *Theory of H. pylori* (1).

However, *Theory of H. pylori* has failed to prove superior to other historical etiological theories but has instead led to additional mysteries. First, its ulcerogenic mechanism remains unknown (18) and it cannot explain 30 (83.33%) of 36 observations/phenomena related to the bacterium itself. Moreover, it has never clarified the roles of gastric acid and Nonsteroidal Anti-inflammatory Drugs (NSAIDs) in ulcer development (19, 20). Second, out of all the 81 observations/phenomena, 45 are unrelated to *H. pylori* (1), making it very difficult for *Theory of H. pylori* to explain the pathogenesis of peptic ulcers. For instance, ulcer lesions in uninfected patients are one of the most challenging issues for *Theory of H. pylori*. Moreover, although *H. pylori* is ubiquitously present in the stomach, gastric ulcers are a sharply circumscribed and located primarily in the gastric antrum and lesser curvature (5, 21), contradicting an infection-based explanation for their morphology and predilection sites. Third, similar to other historical etiological theories, *Theory of H. pylori* has never explained the birth-cohort phenomenon and seasonal variation of peptic ulcers (2, 3), instead introducing an additional epidemiological mystery, the African enigma (17, 22).

Existing data have presented a controversial view of the role of *H. pylori* in peptic ulcers (23–27). Record and Rubin listed four facts to refute that ‘*H. pylori* is a causative agent of peptic ulcers’ (28). Firstly, not all ulcer patients are infected. Secondly, an animal model of *H. pylori*-induced ulcers has never been established. Thirdly, *H. pylori* does not induce gastric acid hypersecretion but rather causes hypochlorhydria in duodenal ulcers. Lastly, bacterial eradication does not cure the disease. In 1995, Rauws and Tytgat concluded that the association between *H. pylori* and peptic ulcers is nonspecific, stating that other factors are required for ulcer development and that a strong association between infection and disease does not necessarily imply causation (17). In 1999, Tovey and Hobsley also disputed the causal role of *H. pylori* in duodenal ulcers, highlighting that in countries with low infection rates, 30–40% of duodenal ulcer patients were *H. pylori* negative, and complete eradication could not prevent relapse (29). Further, Zelickson et al. found only 26% of 128 ulcer patients requiring surgery were infected, further challenging the causal role of the bacterium (30). Evidently, the well-documented *H. pylori*-negative cases challenge Marshall’s claim, creating a paradox: while many cases may be infectious, a substantial subset must be classified as a ‘pathogen-free infectious disease’.

Fortunately, a book titled *Philosophical Principles of Life Science* was published in 2012, proposing a universal law for life science and medicine known as the Complex Causal Relationship (CCR) (31). To test its validity, peptic ulcers were selected as a model disease, leading to the development of a new etiological theory, *Theory of Nodes* (32, 33). Departing from the prevailing *Theory of H. pylori*, this theory hypothesizes that peptic ulcers are a psychosomatic disease triggered by psychological stress, wherein the bacterium may play a secondary role in only the late phase of ulceration (32, 33). A series of six articles (**Supplementary Table S1**) details how this new theory addresses the 15 major characteristics and 81 key observations/phenomena of peptic ulcers, including the long-standing controversies and mysteries such as the birth-cohort phenomenon (34), seasonal variation (35), the roles of gastric acid, *H. pylori*, and NSAIDs (32, 33), and the morphology and predilection sites of gastric ulcers (33). This emerging theory also clarifies the similarities and differences between duodenal and gastric ulcers (33). The first five articles have explicitly elucidated 14 characteristics and 73 observations/phenomena of peptic ulcers (**Supplementary Tables S2**-**S6**) (32–36). This sixth article addresses all the remaining eight observations, including the African enigma (**Supplementary Tables S5**, **S6**). Herein, a novel three-phase psycho-neuropathological model derived from *Theory of Nodes* is introduced to guide the rigorous evaluation of the two competing etiological paradigms: the prevailing *Theory of H. pylori* and the emerging *Theory of Nodes*, aiming to resolve the long-standing controversies surrounding *H. pylori*’s role in peptic ulcers and paving the way for a full resolution of the onset and relapse of the disease.

## 2 Methods

### 2.1 Rationale guiding this analysis

An etiological theory proposing the correct cause should be able to comprehensively elucidate all characteristics, observations/phenomena, controversies, and mysteries of the disease. Therefore, the documented characteristics, observations/phenomena, controversies, and mysteries of peptic ulcers in the literature can serve as a set of empirical benchmarks to impartially evaluate and compare the explanatory power of the prevailing *Theory of H. pylori* and the emerging *Theory of Nodes*. Accordingly, the objective of this comparative analysis is to assess the logical coherence, comprehensiveness, and predictive capacity of the two competing theories’ explanations for these benchmarks.

### 2.2 Literature search strategy

A systematic search of PubMed, Web of Science, and Scopus was conducted to ensure a comprehensive overview of the literature. The search encompassed peer-reviewed journal articles, including randomized trials, observational studies, original articles, reviews, commentaries, and editorials. Books on peptic ulcers and disease pathogenesis were also incorporated. Eligible articles comprised published studies on peptic ulcer research from the past 300 years, covering genetics, anatomy, etiology, psychology, epidemiology, neurology, pathology, bacteriology, clinical statistics, and pre-clinical studies. For etiological theories, only those supported by multiple reproducible clinical, epidemiological, and laboratory observations were retained for in-depth analysis in this study.

### 2.3 Literature selection criteria

Studies examining characteristics or observations/phenomena of peptic ulcers were considered. The search terms covered all known characteristics, such as *H. pylori* infection, gastric acid, epidemiology, morphology, relapse and multiplicity, and NSAIDs. Reference lists of selected studies were also reviewed. For epidemiological surveys, data on varying percentages for rates of *H. pylori* infection, both in patients and the general population, were included. Unreproducible studies were excluded. When multiple studies reported similar findings, priority was given to the earliest publication, as these were deemed foundational for subsequent research.

### 2.4 Data synthesis

The results of the systematic search and selection identified 13 etiological theories, reflecting the historical evolution of studies on peptic ulcers and providing a multidisciplinary view of this disease, along with 15 major characteristics and 81 key observations/phenomena, all reported in reproducible, peer-reviewed literature. The compilation has been published as a data article (1). The collection of 15 major characteristics and 81 key observations/phenomena serves as the set of empirical benchmarks for impartially evaluating the explanatory power of the two competing theories in this analysis. These benchmarks represent reproducible, empirically validated observations that any etiological theory must explain to demonstrate its validity.

### 2.5 Formulating a psycho-neuropathological model as a first principle

The pathogenesis outlined by *Theory of Nodes* (32, 33, 36) is formalized into a three-phase psycho-neuropathological model. This framework integrates the five major historical etiological theories, each substantiated by extensive empirical evidence, encompassing *Psychosomatic Theory* (37), *Stress Theory* (38), *Nerve Theory* (39, 40), ‘*No acid, no ulcer*’ (6), and *Theory of H. pylori* (9). This model serves as a first principle, providing a unified framework to guide all subsequent analyses and to comprehensively explain the 15 major characteristics and 81 key observations/phenomena collated from the literature, including the controversies and mysteries associated with peptic ulcers.

### 2.6 Application of a six-perspective critical analysis framework

Building upon this first-principles model, a six-perspective comparative analysis evaluates the etiological validity of the two competing theories by: 1. examining whether the three lines of supporting evidence truly demonstrate *H. pylori* causation; 2. providing alternative interpretations for observations currently attributed to *H. pylori*; 3. applying historical definitions of ‘etiological factors’ and ‘causality’ for a conceptual evaluation of *H. pylori*’s causal claims; 4. comparing the two theories’ capacity to resolve long-standing epidemiological mysteries; 5. assessing their explanatory power for major peptic ulcer characteristics; and 6. validating them against clinical observations derived from major historical theories. Based on these analyses, the explanatory effectiveness of the two competing theories is synthesized into a summary table, which integrates the findings from all six perspectives into a unified, comprehensive comparison.

## 3 Results

Unlike all the 13 etiological theories derived from empirical data in modern medicine (1), the new theory proposed in the six-article series originated from the application of the CCR and its methodology (32, 33), which guided the integration of the five major historical theories to paint a complete picture of the pathogenesis of peptic ulcers (36). Among all the details described by this new theory, the morphology of gastric ulcers can be explained by the presence of hypothetical submucosal nodes in the gastric wall, which represents the only gap requiring empirical verification. Thus, this new theory was designated as *Theory of Submucosal Nodes*, or briefly, *Theory of Nodes* (32, 33).

### 3.1 The three-phase psycho-neuropathological model derived from *Theory of Nodes*

**Figure 1:**
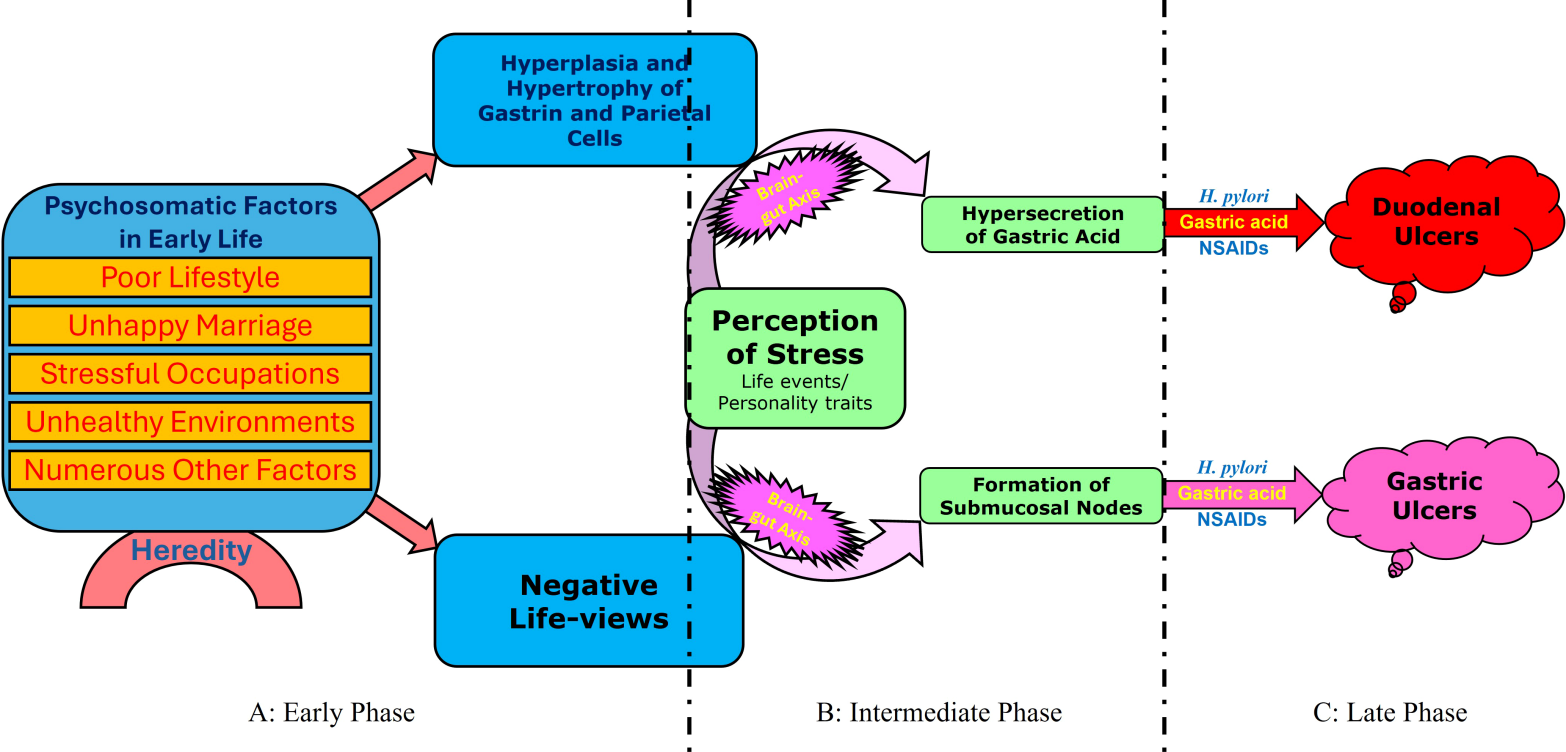
The three-phase psycho-neuropathological model of peptic ulceration. *Theory of Nodes* divides the pathological process of ulceration into three phases. **(A)** Early phase: psychosomatic factors in early life, modulated by hereditary predisposition, either induce the hyperplasia and hypertrophy of gastrin and parietal cells via multiple brain-viscera-endocrine axes or foster a negative life-view. **(B)** Intermediate phase: psychological stress induced by immediate life events or personality traits triggers either gastric acid hypersecretion (leading to duodenal ulceration) or submucosal node formation (leading to gastric ulceration) through brain-gut axis. **(C)** Late phase: Local aggressive factors, primarily gastric acid, *H. pylori*, and NSAIDs, corrode the gastro-duodenal mucosa, leading to ulceration.

Building on the hypothesis, *Theory of Nodes* divides the pathological process of peptic ulceration into three phases (**Figure 1**) (32, 33, 36). In the early phase, psychosomatic factors in early life (such as poor lifestyle, stressful occupations, unhealthy environments, or long-term emotional distress) induce either the hyperplasia and hypertrophy of gastrin and parietal cells in the stomach (for duodenal ulcers) or the development of a negative life-view (for gastric ulcers) via multiple brain-viscera-endocrine axes (32, 33) (**Figure 1A**). During the intermediate phase, psychological stress triggers the core pathophysiological events through the brain-gut axis (39, 41, 42): the hypersecretion of gastric acid (32) or the formation of submucosal nodes, a theoretical construct representing aseptic necrotic tissue in the gastric wall (**Figure 1B**) (33). The late phase is a corrosive process caused by local aggressive factors in the gastro-duodenum, such as gastric acid, *H. pylori*, and NSAIDs, ultimately resulting in peptic ulceration (**Figure 1C**). By establishing a direct, mechanistic link between psychosomatic factors, psychological stress, and gastrointestinal pathology, this three-phase psycho-neuropathological model prioritizes biological plausibility and mechanistic clarity. It thereby provides a foundation to explain the 15 characteristics and 81 observations/phenomena of peptic ulcers, facilitating a comprehensive reevaluation of *H. pylori*’s role in the disease.

### 3.2 In-depth analyses dispute all the three lines of supporting evidence

To understand why *Theory of H. pylori* has failed to elucidate the pathogenesis of peptic ulcers over the past four decades, the three lines of evidence cited by proponents of the infectious model, including Marshall’s original work, are carefully examined herein. If these historical foundations prove inconclusive, then all subsequent research predicated on the *H. pylori*-as-cause hypothesis warrants re-examination.

#### 3.2.1 The underlying mechanism of higher *H. pylori* infection rate in clinical patients

First, the three-phase model elucidates that *H. pylori* is not involved in the early and intermediate phases of peptic ulceration (**Figure 1**). In both *H. pylori*-positive and -negative patients, ulceration is initiated not by *H. pylori* infection but by psychosomatic factors in early life (**Figure 1A**) (32, 33). Thus, *H. pylori* is not a cause but a risk factor that plays a secondary role only in the late phase of peptic ulceration (**Figure 1C**), exacerbating ulcer symptoms, delaying healing, and increasing morbidity and mortality rates (32, 33). Consequently, infected individuals are more likely to seek medical care and become clinical patients due to exacerbated symptoms, while uninfected individuals often remains subclinical with milder symptoms. This skews epidemiological surveys: many *H. pylori*-negative subclinical patients are excluded from the calculation, inflating infection rates among clinical patients (**Table 1**). Therefore, ‘clinical patients have a higher infection rate than the general population (11–14)’ cannot be used to support a causal role of *H. pylori* in peptic ulcers.

**Table 1:** Clinical patients have higher infection rates due to exacerbated symptoms.

Measure	*H. pylori*- (n=100)	*H. pylori*+ (n=100)	Total (n=200)
Actual number of patients	20	20	40
Number of subclinical patients (a)	15	5	20
Number of clinical patients (b)	5	15	20
Clinical morbidity rates	5%	15%	10%
Observed infection rate in clinical patients 75%			
Infection rate in the general population 50%			

The infection rate of *H. pylori* in the general population is assumed to be 50%. 100 individuals were included in each of the *H. pylori*-negative and *H. pylori*-positive groups. This table demonstrates a selection bias where *H. pylori* infection exacerbates ulcer symptoms, making infected individuals more likely to seek medical care and be identified as clinical patients. This leads to the exclusion of subclinical patients (a) from clinical studies. Consequently, the infection rate is calculated only from the clinical patient cohort (b), resulting in a higher observed infection rate among clinical patients (75%) than in the general population (50%).

#### 3.2.2 Larger ulcer lesions and delayed healing do not originate from *H. pylori* infection

Second, larger ulcer lesions and a delayed healing in *H. pylori*-positive patients (15, 16) also do not imply that peptic ulcers are initiated by the infection. As the three-phase model illustrates, all ulcers, regardless of size, are initiated by psychosomatic factors inducing the hyperplasia and hypertrophy of gastrin and parietal cells in the stomach (32) or the development of a negative life-view (**Figure 1A**) (33). Subsequent gastric acid hypersecretion and submucosal node formation in the intermediate phase (**Figure 1B**), merely creates conditions for *H. pylori* to cause larger ulcer lesions and delay healing in the late phase among infected patients (**Figure 1C**) (32, 33). Clearly, despite larger lesions and a delayed healing in *H. pylori*-positive patients, peptic ulceration does not originate from the infection, indicating that these manifestations cannot be used to support the claim ‘a causal role of *H. pylori* in peptic ulcers’.

#### 3.2.3 Does “bacterial elimination reduces relapse rates” support *Theory of H. pylori*?

Third, the observation that *H. pylori* elimination significantly reduces relapse rates was another cornerstone of Marshall’s hypothesis (10). However, if *H. pylori* were the sole and necessary cause, its eradication would be expected to prevent nearly all relapses. The consistent ~20% relapse rate post-eradication (43, 44), which vastly exceeds the long-term reinfection rates as low as ~1% per year in Europe and Australia (11, 45, 46), indicates that relapse cannot be attributed solely to reinfection but implicates ‘other factors’ (17). *Theory of Nodes* provides a coherent explanation: it proposes that *H. pylori* plays a secondary role predominantly in the late phase of ulceration (**Figure 1C**). Consequently, eradication therapy does not address the primary triggers, namely, the preexisting hyperplasia and hypertrophy of gastrin and parietal cells or the underlying negative life-view (**Figure 1A**). These primary factors, which precisely constitute the ‘other factors’, can reactivate acid hypersecretion and node formation, leading to relapse. Within this framework, duodenal ulcers occur when the total corrosive intensity of all aggressive factors, including gastric acid, *H. pylori*, and NSAIDs, exceeds mucosal resistance (32). *H. pylori* eradication substantially reduces this intensity in infected individuals, thereby preventing relapses in ~80% of cases. Thus, the high efficacy of eradication therapy is consistent with *H. pylori* being an important but not essential contributing factor in the late phase, and therefore, the clinical finding that ‘bacterial elimination significantly reduces relapse rates (10, 11, 17)’ cannot be used to support the claim that ‘peptic ulcers are an infectious disease caused by *H. pylori*’.

Collectively, all three lines of evidence, while underscoring the clinical significance of *H. pylori* infection and the benefits of eradication, do not prove a causal relationship between *H. pylori* infection and peptic ulcers. Instead, they are logically consistent with the perspective that *H. pylori* is not the cause but plays a secondary role in only the late phase of peptic ulceration, exclusively exacerbating symptoms, delaying healing, and increasing clinical morbidity.

### 3.3 Alternative interpretations for two of three observations attributed to *H. pylori*

In addition to the three lines of supporting evidence, *Theory of H. pylori* can explain three additional observations. For two of these three, however, *Theory of Nodes* provides alternative and arguably more comprehensive explanations, suggesting a potential misinterpretation within the *H. pylori* paradigm.

In a relatively isolated group of Australian Aboriginals, peptic ulcers were rare (47). Similarly, a two-year study of Pima Indians found no ulcer cases (48), whereas 10% of the contemporary Caucasian population in North America developed ulcers (49). *Theory of H. pylori* attributes these differences to the low prevalence of *H. pylori* among Pima Indians and Australian Aboriginals and the high infection rates in Caucasians. However, *Theory of Nodes* elucidates that the low incidence of peptic ulcers in indigenous groups was due to a pastoral lifestyle with fewer social conflicts and therefore, they were leading a less stressful life (**Figures 1A, B**), while the higher incidence in Caucasians resulted from modern societal pressures like financial crises, unemployment, or competition (**Figures 1A, B**) (32, 33). *Theory of H. pylori* can explain a third observation that ‘*H. pylori* infection appears to reduce ulcer development in NSAIDs users (17)’, but this observation alone is insufficient to support the claim ‘peptic ulcers are a *H. pylori*-caused infectious disease’.

Taken together, although *Theory of H. pylori* offers an explanation for six of the 36 observations associated with the bacterium itself, it leaves the majority unexplained. Furthermore, when interpreted within the three-phase model illustrated in **Figure 1**, a more comprehensive etiological framework, the six explainable observations do not provide conclusive evidence for ‘peptic ulcers are an infectious disease caused by *H. pylori*’. Consequently, it is in fact difficult to find supporting evidence for the prevailing *H. pylori*-as-cause hypothesis.

### 3.4 Definitions of ‘etiological factor’ and ‘causality’ dispute the causal role of *H. pylori*

*Theory of Nodes* has systematically addressed the 36 *H. pylori*-associated observations in a series of six articles (**Supplementary Table S6**), suggesting that the conventional understanding of *H. pylori*’s role in peptic ulcers warrants re-evaluation. This comprehensive explanation raises a fundamental question regarding etiological classification: can a factor whose involvement is confined to the late phase of a disease process be considered its primary cause? To address this question, key historical definitions of ‘etiological factor’ and ‘causality’ are briefly reviewed and applied here.

#### 3.4.1 Examining *Theory of H. pylori* by Hill’s nine criteria (1965)

In 1965, Hill proposed nine criteria to assess causality: strength of association, consistency, specificity, temporality, biological gradient, plausibility, coherence, experimental evidence, and analogous evidence (50). These criteria have been widely used to evaluate causal relationships between potential factors and disease (51–54). The high prevalence of *H. pylori*-negative cases (~20% duodenal, ~50% gastric ulcers) (16, 20, 44, 55–57) challenges whether the strength of association, consistency, specificity, and coherence are sufficient to support ‘a causal role of *H. pylori* in peptic ulcers’. For simplicity, this analysis focuses only on ‘temporality’, a key criterion requiring that ‘a cause must precede its effects’ (50). Notably, *H. pylori* infection neither initiates the disease as psychosomatic factors in early life nor precedes gastric acid hypersecretion (duodenal ulcers) and submucosal node formation (gastric ulcers) (**Figures 1A, B**). Instead, it acts secondarily in only the late phase of ulceration (**Figure 1C**) (32, 33), which is inconsistent with the established definitions of ‘etiological factor’ and ‘causality’ of a disease.

#### 3.4.2 Evaluating *Theory of H. pylori* by historical definitions of etiology

In 1986, Wulff defined etiology as ‘a linear process where upstream causes represent the etiological factors and the causal intermediates represent the pathological process’ (58, 59). When this definition is applied, *H. pylori* is neither an upstream cause nor a causal intermediate but plays a secondary role downstream in the ulceration process (**Figure 1**). Susser added that ‘the cause must precede the effect and occur together with the putative effect’ (60). However, *H. pylori* infection precedes neither abnormal neurotransmitter levels in the brain as shown in neurological studies (39, 40) nor consistently co-occurs with ulcers, given ~20% duodenal and ~50% gastric ulcer patients are *H. pylori* negative (16, 20, 44, 55–57). Witthöft further argued that ‘only causes directly initiating the disease process (thus necessarily preceding it) are etiological factors’ (61). Given that *H. pylori* does not initiate the disease but plays a fortuitous, secondary role in the late phase (**Figure 1C**), its function does not align with the established definitions of ‘etiological factor’ and ‘causality’.

Based on these analyses, the proposed causal relationship between *H. pylori* and peptic ulcers is difficult to reconcile with core scientific definitions. Current understandings of ‘etiology’ and ‘causality’ do not accommodate the claim that ‘*H. pylori* is an etiological factor of peptic ulcers’. A robust causal conclusion typically requires a comprehensive understanding of the upstream, midstream, and downstream processes in disease development. The causal role of *H. pylori* was concluded prior to the elucidation of this full pathogenic sequence. In contrast, the novel three-phase model explicitly delineates the early (upstream), intermediate (midstream), and late (downstream) phases of ulceration. This model clearly positions *H. pylori*’s involvement exclusively in the late phase/downstream (**Figure 1C**), which is inconsistent with the characteristics of a primary causative agent. It is therefore understandable that *Theory of H. pylori* has been associated with numerous unresolved controversies and mysteries.

### 3.5 Epidemiological observations dispute the causal role of *H. pylori* in peptic ulcers

If peptic ulcers are an infectious disease caused by *H. pylori*, then the hypothesis should be able to fully elucidate all epidemiological phenomena associated with the disease. However, the infectious model fails to explain not only the three major epidemiological observations but also numerous other regular investigations. This persistent failure further indicates that *H. pylori* cannot be considered an etiological agent of peptic ulcers.

#### 3.5.1 Challenging *Theory of H. pylori* with three major epidemiological mysteries

*Theory of H. pylori* has never adequately explained the three major epidemiological mysteries of peptic ulcers: the birth-cohort phenomenon (2), seasonal variation (3), and African enigma (17, 22). Sonnenberg’s mathematical model based on *H. pylori* failed to explain the time lag between gastric and duodenal ulcers (62, 63), along with several other issues. Moreover, the seasonal prevalence of *H. pylori* infection does not align with ulcer seasonality (64, 65). This misalignment prevents *Theory of H. pylori* from linking seasonal changes to disease variation, thereby leaving the seasonality of peptic ulcers without a mechanistic explanation within its framework. The African enigma, characterized by a high *H. pylori* prevalence but variable ulcer morbidity rates in African countries (17, 22), also remains unresolved. Attempts to explain these phenomena via *H. pylori* mutations, such as the distinction between cag- and cag+ strains (66–70), have failed to resolve these mysteries and in some cases, lead to implausible conclusions. For instance, explaining the birth-cohort phenomenon with the mutation theory would require *H. pylori* to mutate into a more virulent strain (cag+) at the start of World War I and revert to a less virulent strain (cag-) at the end of World War II, whereas explaining the seasonal variation and African enigma would demand the bacterium to mutate seasonally and regionally. Clearly, such extrapolations violate the laws of natural bacterial evolution and are thus untenable.

In contrast, guided by the three-phase model, *Theory of Nodes* readily and comprehensively elucidates the three major epidemiological mysteries. Crucially, the birth-cohort phenomenon and seasonal variation are explained without invoking *H. pylori* infection, directly indicating the bacterium is not causative (32–36). In *Theory of Nodes*, environmental factors induce psychological stress during the intermediate phase, triggering ulceration (**Figure 1B**). Each environmental factor produces its own mortality/morbidity fluctuation curve, with psychological impacts dominating the increasing and decreasing trends (32, 33). Integration of these curves for all individual environmental factors simulates the patterns of the birth-cohort phenomenon and seasonal variation, revealing that both mysteries are the holistic effects of the psychological impacts of multiple environmental factors (32, 33). However, after integration, the increasing and decreasing trends caused by an individual environmental factor are obscured by other factors, thereby rendering the detectable causal role of each factor invisible in the overall fluctuation curves and ultimately resulting in the two epidemiological mysteries. Similarly, the regional differences in morbidity rates across Africa stem not from *H. pylori*, but from varied and stressful environmental factors, such as political stability, economic development, and social welfare disparities. The default assumption that ‘*H. pylori* is the cause of peptic ulcers’ gave rise to the third epidemiological mystery, the African enigma (17, 22). When considered together, the three major epidemiological mysteries provide compelling evidence that peptic ulcers are not an infectious disease caused by *H. pylori*, but a psychosomatic disease triggered by psychological stress.

#### 3.5.2 Other epidemiological observations disputing the causal role of *H. pylori*

Beyond these mysteries, substantial epidemiological observations also present challenges to *Theory of H. pylori*. For instance, only 27% of symptomatic children with peptic ulcers in the United States are *H. pylori* positive (71), a finding difficult to reconcile with the bacterium as the primary cause. This picture is further complicated by the low incidence of ulcers in children despite high infection rates (71 ,72). Additionally, infection rates are less than 50% in gastric ulcer patients (20, 44), with up to 40% of duodenal ulcer patients being *H. pylori*-negative in some countries (29) and approximately 20% of peptic ulcers showing no association with the bacterium in Poland (16). Autopsy studies revealing that 20-29% of males and 11-18% of females had unknowingly suffered from ulcers (73–75), alongside the higher incidence of duodenal ulcers in large cities compared to rural areas (76, 77), further introduce an epidemiological pattern not readily explained by the prevailing *Theory of H. pylori*.

In contrast, these observations find a coherent explanation within the three-phase psycho-neuropathological model, which posits that peptic ulcers are not an infectious disease caused by *H. pylori* but a psychosomatic disease triggered by psychological stress (32, 33), thereby providing a comprehensive explanation for these epidemiological data. According to this model, only individuals with hyperplasia and hypertrophy of gastrin and parietal cells or a negative life-view (**Figures 1A, B**) are predisposed to developing ulcers (32, 33). This explains the high prevalence of *H. pylori* infection alongside the low incidence of peptic ulcers. The high percentage of *H. pylori*-negative ulcer patients (29, 55) further supports that *H. pylori* is not the cause. Younger patients, with fewer cumulative life events due to their short lifetimes, are less likely to develop ulcers. Children with hyperplasia and hypertrophy of gastrin and parietal cells or a negative life-view are rare, explaining the low incidence in this age group. Gender disparities in ulcer prevalence reflect historical societal roles: from the 1900s to the 1950s, men were more likely to engage in social conflicts and bear economic pressure than women, consistent with autopsy reports showing higher ulcer prevalence in males. Additionally, urban environments foster more stressful lives with intensive social competition (78, 79), accounting for the higher incidence of peptic ulcers in large cities than in rural areas.

### 3.6 Characteristics of peptic ulcers dispute the causal role of *H. pylori*

Of all the 15 major characteristics, 14 show no established necessary link to *H. pylori* (1). Regarding the single characteristics specific to *H. pylori*, the prevailing *Theory of H. pylori* does not provide a consistent explanation for 30 (83.33%) of the 36 *H. pylori-*associated observations. In contrast, when analyzed through the core premise of the three-phase model, in which peptic ulcers are a psychosomatic disease triggered by psychological stress, *Theory of Nodes* offers a complete and coherent explanation for the 15 characteristics and the 36 associated observations (**Supplementary Tables S2**, **S5**, **S6**).

#### 3.6.1 Inconsistencies in explaining the predilection sites and morphology of gastric ulcers

First, *Theory of H. pylori* has never explained the predilection sites and morphology of gastric ulcers. Although the density of *H. pylori* is significantly higher in the incisura angularis on the lesser curvature than in the gastric antrum (80), gastric ulcers occur most often in the gastric antrum, not the lesser curvature. Moreover, since *H. pylori* is omnipresent throughout the stomach, all regions should be susceptible to ulceration. However, gastric ulcers consistently localize to the gastric antrum and lesser curvature, exhibiting a characteristic ‘hole-punch’ appearance with clean edges, as if cut by a knife (5, 21). In contrast, guided by the three-phase model, *Theory of Nodes* provides a clear explanation: it posits that the predilection sites are determined by the location of submucosal nodes, which are influenced by nerve density in the stomach (33). The gastric antrum and lesser curvature contain the largest ganglia and highest density of nerve plexus (81), suggesting they receive more pathogenic nerve impulses from the Central Nervous System (CNS) than other parts of the stomach. This local neuroanatomy makes them the predilection sites for gastric ulcers. Furthermore, the spherical shape of submucosal nodes accounts for the characteristic ‘hole-punch’ morphology of gastric ulcers (33).

#### 3.6.2 Failure to explain the relapse, multiplicity, and self-healing of peptic ulcers

Another core challenge for *Theory of H. pylori* is its inability to explain ulcer relapse, multiplicity, and self-healing. If *H. pylori* is the sole cause, its eradication should effectively prevent relapses. Yet, ulcers often relapse after successful eradication (43, 82–84). *Theory of Nodes* clarifies this by positing that as long as the underlying psychosomatic factors and psychological stress persist, ulcer relapses are inevitable, giving rise to the adage ‘*Once an ulcer, always an ulcer*’ (85). *Theory of Nodes* further proposes that targeting these root causes, early-life psychosomatic factors and immediate psychological stress, can significantly reduce disease onset and effectively prevent relapses (34, 36), representing a true etiological therapy beyond anti-*H. pylori* treatments. Regarding multiplicity, *Theory of Nodes* attributes the number of concurrent ulcers to the distribution of submucosal nodes (33), which is governed by individual gastric neuroanatomy. While ulcers can heal spontaneously through tissue regeneration, this natural process can be impeded by local aggressive factors, including gastric acid, *H. pylori*, and NSAIDs.

#### 3.6.3 Inability to explain the bleeding and perforation of peptic ulcers

*Theory of H. pylori* is further undermined by its failure to elucidate the bleeding and perforation of peptic ulcers. For instance, studies show a lower prevalence of *H. pylori* infection in patients with these complications (86–88). In contrast, *Theory of Nodes* provides a clear explanation: bleeding or perforation occurs when patients experience severe psychological stress, leading to excessive acid secretion or the formation of larger submucosal nodes (**Figure 1B**). Bleeding follows vessel rupture near ulcers, while perforation arises if nodes erode deeply into the serosa (33). In contrast, patients without these complications are typically less affected by psychological stress, but a co-existing *H. pylori* infection exacerbates symptoms, prompting them to seek clinical care. This explains the higher *H. pylori* infection rate in this group compared to those with complications, as outlined in **Table 1**.

#### 3.6.4 How does *Theory of H. pylori* distinguish gastric ulcer from duodenal ulcer?

Another fundamental limitation of *Theory of H. pylori* is its inability to distinguish gastric from duodenal ulcers, despite their being genetically, etiologically, and epidemiologically different two diseases (5, 89). For example, it cannot explain why stress induces only gastric ulcers in lab settings, or how chemicals cause duodenal ulcers in uninfected rats (90). In decisive contrast, *Theory of Nodes* proposes that patients with gastric versus duodenal ulcers represent two genetically distinct populations, yet both are psychosomatic diseases triggered by psychological stress that generates abnormal neurotransmitters in the CNS, resulting in pathogenic nerve impulses to the stomach (36). While the stomach is the effector organ for both diseases, the outcomes differ: duodenal ulcers result from acid hypersecretion (32), whereas gastric ulcers arise from submucosal node formation (33). Thus, controlling acid secretion is essential only for duodenal ulcers. Critically, *H. pylori* and NSAIDs play secondary roles in only the late phase (**Figure 1C**) and are thus not essential for either duodenal or gastric ulcers. *Theory of Nodes* further clarifies that acute stress, which can be reproduced in laboratory animals, directly causes gastric ulcers, whereas chronic stress, which requires approximately five years to induce hyperplasia and hypertrophy of gastrin and parietal cells, cannot be simulated in laboratory settings (32, 34). This chronic pathological process explains the difficulty in establishing animal models for duodenal ulcers (28, 90). Chemicals like cysteamine and propionitrile bypass this chronic process by interacting directly with the effector organs of pathogenic nerve impulses (**Figure 1B**), triggering acid hypersecretion and ultimately resulting in duodenal ulcers (91, 92). This experimental evidence indicates that *H. pylori* infection, despite its high prevalence in many regions, does not appear to be an essential factor for duodenal ulceration.

#### 3.6.5 Why do only duodenal ulcers correlate strongly with *H. pylori* density?

A striking epidemiological inconsistency arises from the fact that only ~50% of gastric and 80% of duodenal ulcer patients are infected with *H. pylori* (16, 20, 44, 55–57), a fact that led to listing the infection as a characteristic of peptic ulcers (1). However, *Theory of H. pylori* cannot explain why duodenal ulcer presence correlates strongly with *H. pylori* density, while the bacterium’s association with gastric ulcers remains less clear (93). Guided by the three-phase model (**Figure 1C**), *Theory of Nodes* resolves this paradox. It posits that duodenal ulcers occur only when the total corrosive intensity from all local aggressive factors (gastric acid, *H. pylori*, and NSAIDs) exceeds duodenal mucosal resistance. Therefore, higher *H. pylori* density contributes to a greater corrosive intensity, directly explaining the close association with duodenal ulcers (32). In contrast, gastric ulcers are driven primarily by submucosal node formation, not corrosion from local aggressive factors (**Figure 1B**), accounting for the weaker link to *H. pylori* infection (33).

#### 3.6.6 Animal models relying solely on *H. pylori* have never been established

Finally, animal models, a cornerstone of causal inference, have failed to provide support for *Theory of H. pylori*. Since the three-phase model posits that *H. pylori* is not an etiological factor (**Figure 1**), *Theory of Nodes* predicts that animal models relying solely on *H. pylori* cannot be established. Consistent with this prediction, *H. pylori* infection in rats induced only mild to moderate mucosal inflammation without ulcers; oxyntic mucosa ulcers that occurred were equally present in infected and uninfected groups and were solely attributable to acetic acid exposure on the serosal side (15). Most conclusively, Dr. Marshall, the discoverer of *H. pylori*, conducted a self-experiment in 1984 by drinking a concoction made from cultured *H. pylori*. He expected to develop an ulcer, potentially years later, but instead only experienced gastritis, which was later cured with antibiotics (94). This outcome, gastritis without ulcer formation, is inconsistent with the prediction that *H. pylori* is the primary cause of peptic ulcers.

### 3.7 Historical observations dispute the causal role of *H. pylori* in peptic ulcers

Although none of the 13 historical etiological theories explained the pathogenesis of peptic ulcers, five of them (*Psychosomatic Theory*, *Stress Theory*, *Nerve Theory*, ‘*No acid, no ulcer*’, and *Theory of H. pylori*) were substantiated by extensive clinical, epidemiological, and laboratory observations, contributing critical discoveries (32). Notably, observations underpinning these five major theories provide a rigorous framework for evaluating the two competing theories, *Theory of H. pylori* and *Theory of Nodes*.

#### 3.7.1 Challenges from psychosomatic and psychological research

*Theory of H. pylori* cannot elucidate the observations supporting *Psychosomatic Theory* (95) and *Stress Theory* (38). Feldman et al. found a strong correlation between psychosocial factors and ulcer disease, with patients exhibiting significantly more emotional distress, such as depression and anxiety (96). Additionally, stressful life events frequently preceded ulcer onset, and symptoms often subside once the stressors are resolved (97, 98). In stark contrast, *Theory of Nodes*, guided by the three-phase model, positions psychosomatic factors and psychological stress as upstream events in the early phase (**Figure 1A**), while *H. pylori* is relegated to a secondary, downstream role in the late phase (**Figure 1C**). This hierarchical distinction clarifies why a downstream factor cannot influence upstream processes, explaining the failure of *Theory of H. pylori* to explain these observations and further supporting that the bacterium is not the cause of peptic ulcers.

#### 3.7.2 Pitfalls when confronting findings from neurological studies

*Theory of H. pylori* cannot explain the findings supporting *Nerve Theory* (99), which posits that peptic ulcers are a ‘*brain-driven*’ event (39, 40). Research shows the CNS is activated in animals exposed to ulcerogenic stress (39, 40, 100, 101). For instance, pretreatment with intraperitoneal EDTA (a calcium chelator) or CaCl_2_ significantly reduced or increased cold stress-induced gastric ulcers, respectively, linking ulceration to elevated calcium/calmodulin-dependent catecholamine synthesis in the brain (42). Furthermore, the amygdala modulates stress-induced gastric erosions (39, 102, 103), and manipulation of its central nucleus can produce gastric ulcers (39, 41, 103, 104). In contrast, *Theory of Nodes* interprets these neurological findings as manifestations of the intermediate phase (midstream) of peptic ulceration (**Figure 1B**), while *H. pylori* acts secondarily in the late phase (downstream) (**Figure 1C**). Since the midstream process is independent of downstream infection, it is not surprising that *Theory of H. pylori* cannot elucidate many of the neurological research findings.

#### 3.7.3 How can *Theory of H. pylori* explain gastric acid-associated observations?

Duodenal ulcer patients exhibit increased basal and maximal acid secretion (105, 106), whereas most gastric ulcer patients are normo- or hypo-secretors (107, 108). This supports Schwartz’s dictum ‘*No acid, no ulcer*’ (6), which holds true primarily for duodenal ulcers (106). Notably, *Theory of H. pylori* has never addressed this secretory difference (97). If *H. pylori* were the most important etiological factor for duodenal ulcers as Marshall claimed (10), its infection should cause acid hypersecretion. However, the infection actually suppress acid secretion, increasing the pH of gastric juice (97), and consistent hypersecretion post-infection has never been observed (109, 110). In decisive contrast, *Theory of Nodes* proposes that acid hypersecretion stems not from *H. pylori* but from psychosomatic factors in early life, causing the hyperplasia and hypertrophy of gastrin and parietal cells in the stomach (**Figure 1**) (32). The three-phase model also clarifies that not *H. pylori*, but increased acid secretion dominates local features of duodenal ulcers (**Figure 1B**) (32). Conversely, for gastric ulcers, the local features are dominated not by aggressive factors in the stomach but by the submucosal nodes in the gastric wall (**Figure 1B**). This explains why most gastric ulcer patients are normo- or hypo-secretors of gastric acid.

#### 3.7.4 Failure to explain the vast majority of the observations on the bacterium itself

The most compelling evidence for the limitations of *Theory of H. pylori* is its failure to elucidate 30 (83.33%) of 36 observations/phenomena associated with the bacterium itself (1). In contrast, guided by the three-phase model, which posits that *H. pylori* is not an etiological factor but rather a secondary, non-essential aggressive factor, *Theory of Nodes* provides a complete explanation for the 36 *H. pylori*-associated observations/phenomena (**Supplementary Table S6**). The analysis clarifies that the prevailing *Theory of H. pylori* misinterpreted the clinical significance of the bacterial infection and eradication (as reflected in the three lines of supporting evidence) as proof of a causal relationship between *H. pylori* and peptic ulcers (**Table 1**). Moreover, two of the three epidemiological observations attributed to the bacterium may also be misinterpreted. Crucially, 45 observations/phenomena wholly unrelated to the bacterium (1) definitively demonstrate *Theory of H. pylori*’s inability to elucidate the pathogenesis of peptic ulcers. In light of this comprehensive analysis, the statement that ‘a causal role of *H. pylori* in peptic ulcers is supported by clinical and epidemiological data’ calls for much deliberation. **Table 2** presents a concise summary of both supporting and opposing evidence regarding the role of *H. pylori* in peptic ulcers, drawn from the existing literature, providing a transparent overview.

**Table 2:** Summary of supporting and opposing evidence on causal claim of *H. pylori*.

Category	Supporting evidence	Opposing evidence
Clinical	1. Higher infection rates in ulcer patients (12–14). 2. Elimination significantly reduce relapse (10, 11, 17). 3. *H. pylori* infection reduces ulcer development in NSAIDs users (17).	1. Relapse, multiplicity, and self-healing unexplained. 2. Bleeding and perforation unexplained. 3. 20-50% patients have no infection (16, 20, 44, 55–57), causing a pathogen-free paradox.
Laboratory	4. Larger ulcers and delayed healing in infected rats; both reversed by elimination (15, 16). 5. There is a dose effect between *H. pylori* and duodenal ulcers only (17, 93).	4. Animal models for *H. pylori*-alone-induced ulcers have never been established (28, 90). 5. The association (dose-effect) between *H. pylori* and gastric ulcers is less clear (17, 93). 6. Paradoxes arise when attempting to explain the morphology and predilection sites of gastric ulcers. 7. Stress-related gastric ulcers are 'brain-driven' events (39, 40).
Epidemiology	6. Peptic ulcers were rare in Australian Aboriginals (47) and Pima Indians (48) due to low *H. pylori* infection rates. 7. The high percentage (10%) of ulcer patients in Caucasian population due to high *H. pylori* infection rates (49).	8. African Enigma (17, 22). 9. Birth-cohort phenomenon and seasonal variation explained without involvement of *H. pylori* (34, 35). 10. Urban-rural ulcer prevalence differences unexplained (76, 77).
Causal Testing		11. Fails Hill’s nine criteria in 1965 (50). 12. Fails Wulff’s definition of etiology in 1986 (58).
Mechanistic	How *H. pylori* causes peptic ulcerations remains unknown (23–27).	13. Similarity and Differences between gastric and duodenal ulcers have never been understood. 14. A synthesis of five major historical theories potentially viable as demonstrated in this study.

The asymmetry between supporting and opposing evidence reflects the actual distribution of observations in the literature, as detailed in Sections 3.2–3.7 and the **Supplementary Materials**.

Synthesizing these lines of evidence, *Theory of Nodes* integrates all the five major historical etiological theories (including Marshall’s *Theory of H. pylori*) into a unified etiological framework. Its three-phase model provides a comprehensive framework for understanding the pathogenesis of peptic ulcers (**Supplementary Tables S2**–**S6**) and explicitly acknowledges the critical contributions from *Psychosomatics Theory*, *Stress Theory*, *Nerve Theory*, and ‘*No acid, no ulcer*’ to a comprehensive understanding of the disease. However, over the past four decades, these foundational discoveries have been largely overshadowed by *Theory of H. pylori*, which conceptualizes peptic ulcers as an infectious disease.

### 3.8 Comparative explanatory power of *Theory of H. pylori* and *Theory of Nodes*

Collectively, the preceding analyses indicate that the claim ‘peptic ulcers are an infectious disease caused by *H. pylori*’ may have overstated the bacterium’s role in the disease (83). Consequently, none of the 15 major characteristics have been fully understood, and the majority of the 81 key observations/phenomena have remained unexplained for four decades. The focus on this single etiological model has coincided with the persistence of numerous controversies and unresolved mysteries of the disease. In contrast, the three-phase model, which conceptualizes peptic ulcers as a psychosomatic disease triggered by psychological stress, offers a framework capable of coherently elucidating the 15 major characteristics and 81 key observations/phenomena of peptic ulcers, including previously unresolved controversies and mysteries (**Supplementary Tables S2**–**S6**). A systematic comparison of the explanatory power of *Theory of H. pylori* and *Theory of Nodes* is summarized in **Table 3**. Thus, the empirical foundation for this comparative analysis consists of 15 major characteristics and 81 key observations curated from the existing literature over centuries. These observations, detailed across six tables, demonstrate the comprehensive explanatory power of *Theory of Nodes*. Evidently, *Theory of Nodes* is supported not only by its systematic approach, but also by the comprehensive explanation of these empirical observations.

**Table 3:** Comparative effectiveness of *Theory of H. pylori* and *Theory of Nodes*.

Etiological theory	*Theory of H. pylori*	*Theory of Nodes*
**15 characteristics**	None could be explained.	The full set is explained.
Etiology	Peptic ulcers are an infectious disease caused by *H. pylori*.	Peptic ulcers are a psychosomatic disease triggered by psychological stress (32, 33).
Morphology of gastric ulcers	Remains unknown	Explained (the only one requires validation) (33).
Predilection sites of gastric ulcers	Remains unknown	Explained (33).
Relapse and Multiplicity	Remains unknown	Explained (33).
Bleeding and Perforation	Remains unknown	Explained (33).
Epidemiology	Failed to explain most epidemiological data. Addressed only 3/6 associated observations, likely reflecting correlation, not causation.	Explains the full set of epidemiological patterns. Re-interprets the three lines of supporting evidence as non-causal correlations.
**81 observations/phenomena**	75 of 81 were not explained.	The full set of 81 is explained
36 observations/phenomena associated with *H. pylori*	30 of 36 were not explained.	The full set of the 36 is explained.
45 observations/phenomena not associated with *H. pylori*	None were explained because unrelated to the bacterium.	The full set of the 45 is explained.
**Four controversies**	None were addressed	The full set is addressed
Roles of *H. pylori*	Controversial.	Addressed: a secondary role in the late phase, not an etiological factor.
Roles of gastric acid	Unknown	Addressed (32, 33).
Roles of NSAIDs	Unknown	Addressed (32, 33).
Idiopathic peptic ulcers	Unknown	Addressed (32, 33).
**Three Major Mysteries**	None were resolved.	All three are resolved.
Birth-cohort phenomenon	Remains mysterious.	Resolved (34).
Seasonal variation	Remains mysterious.	Resolved (35).
African enigma	Remains mysterious.	Resolved.
**Similarities and differences between gastric and duodenal ulcers**	Remains unknown.	Systematically illustrated (33).
**Therapy/effect**	Antiacid and antibiotics treatments were primary therapy; relapse frequently.	Etiological therapy: targeting psychosomatic and psychological roots effectively prevents relapse (34, 36).
**Summary**	1. Failed to explain the pathogenesis of peptic ulcers. 2. Could not explain 15 major characteristics and 75 of 81 observations/phenomena. 3. Was inferior to historical theories; generated more controversies and mysteries.	1. Explains the pathogenesis of peptic ulcers. 2. Explains the full scope of the characteristics, observations, controversies, and mysteries. 3. Refutes *H. pylori* as a causal factor; reclassifies it as a secondary factor.

Values in bold denote major categorical divisions within the table.

## 4 Discussion

### 4.1 *Theory of Nodes* as a superior etiological framework

A valid etiological theory must explain all characteristics and observations/phenomena of the disease. Though *Theory of H. pylori* gradually became the prevailing consensus since the 1980s, its explanatory power has remained stagnant for 40 years, having advanced only marginally beyond six initial observations while leaving all 15 major characteristics and 75 key observations/phenomena unresolved. In contrast, this study demonstrates that the full set of the 15 characteristics and 81 observations/phenomena can be explained if peptic ulcers are recognized as a psychosomatic disease triggered by psychological stress. The superior explanatory power (**Table 3**) suggests that *Theory of Nodes* may have identified the underlying etiology of peptic ulcers, where *H. pylori* is not the cause but a secondary, nonessential risk factor. The prolonged stagnation under the prevailing model may be understood through the lens of a scientific misconception, a framework that appears convincing and persuasive based on initial evidence, but it is ultimately founded on a misinterpretation that diverts research from more productive paths. The failure to explain core disease characteristics for decades, despite its widespread acceptance, fulfills this definition. Recognizing *Theory of H. pylori* as such a misconception clarifies why it has been unable to explain any of the 15 major characteristics and 75 (92.6%) of 81 key observations/phenomena for 40 years, while more controversies and mysteries have accumulated. This recognition not only marks a paradigm shift in ulcer research but also provides invaluable insights for understanding other diseases.

### 4.2 The systematic approach identified two significant methodological oversights

The systematic approach employed in this study facilitated an extensive review of literature beyond peptic ulcers, culminating in the identification of two significant oversights in modern ulcer research. First, Hill’s criteria for causality in 1965 (50), established 22 years before Marshall’s hypothesis, have never been rigorously applied to critically re-evaluate “a causal role of *H. pylori* in peptic ulcers”, despite their extensive use in other diseases (51–54). Second, Wulff’s definition of etiology in 1986 (58), published one year before Marshall’s claim and much earlier than it became the prevailing consensus in the early 2000s, stipulates that the upstream, midstream, and downstream processes of a disease must be elucidated before a causal relationship can be established. Yet, how *H. pylori* causes peptic ulcers remains unknown even today, indicating that the causal conclusion was drawn without fulfilling this prerequisite. Evidently, departure from Hill’s criteria and Wulff’s definition is a principal reason for the *H. pylori* misconception. This 40-year-old misconception, which originated in the late 20th century and still persists today, may continue to serve as a cautionary focus for scholarly attention for centuries to come, warranting its designation as ‘A Cross-century Misconception’. This long-standing interpretation, which was reasonable given the evidence available in the 1980s, can now be re-evaluated as a historical misconception in light of the CCR framework proposed in 2012.

### 4.3 The three-phase model identified the roles of the key factors associated with peptic ulcers

The three-phase psycho-neuropathological model, derived from applying the CCR with its methodology (32, 33), clearly delineates the roles of the key factors associated with peptic ulcers. These factors act in the pathogenesis in a chronological sequence: psychosomatic factors function in the early phase, resulting in hyperplasia and hypertrophy of gastrin and parietal cells or negative life-views. Although these pathogenic changes do not manifest as overt disease, they are prerequisites for later disease development, representing the upstream process of ulceration. Psychological stress is induced in the intermediate phase, acting as a trigger via the ‘brain-gut axis’. Despite its highly transient nature, this phase is an indispensable midstream process. *H. pylori*, gastric acid, and NSAIDs appear in the late phase, which aligns precisely with Wulff’s definition of a downstream process. Evidently, the three-phase model identifies *H. pylori* not as an etiological factor, but as a non-essential risk factor playing a secondary role in only the late phase of ulceration. Significantly, this role identification elucidated the 36 bacterium-associated observations (**Supplementary Table S6**), demonstrating *Theory of Nodes* has revealed the truth: *H. pylori* is not the cause of peptic ulcers, a finding that challenges the prevailing *Theory of H. pylori*.

### 4.4 The elucidated pathogenesis uncovered an overlooked psychosomatic origin for diseases

The pathogenesis of peptic ulcers, as illustrated in the three-phase model, provides a definitive psychosocial-neuropathological framework for how psychosomatic factors cause disease. This is not a correlation, but an elucidated causal pathway: early life adversity and chronic stress operate via brain-body axes to create a pre-disease state, a physical vulnerability in the stomach or a psychological vulnerability in the form of negative life-views. This model demonstrates that the impacts of psychosomatic factors are not transient; they are cumulative and persistent, becoming biologically embedded as minimal pathological changes that amplify an individual’s reaction to later psychological stress, ultimately triggering ulceration. The implications of this psychosomatic model extend far beyond gastroenterology. It provides a mechanistic template for understanding the ‘abstract early phase’ of many diseases, a phase mandated by the CCR but largely invisible to modern medicine. For example, in cancer or AIDS, one can hypothesize that numerous psychosomatic factors similarly wear down the body’s anti-cancer or anti-viral defenses over years or decades. This gradual erosion of innate disease resistance in the early phase would permit the accumulation of carcinogens and gene mutations or the establishment of stable infections in the intermediate phase, ultimately culminating in overt disease. The prevailing neglect of this psychosomatic origin has profound consequences. By focusing research almost exclusively on the intermediate and late phases and their proximal agents (like *H. pylori*, carcinogens and mutations, or HIV), modern medicine has systematically overlooked the foundational, predisposing causes. Consequently, the root cause of most diseases remains unidentified, our understanding is incomplete, and the development of true etiological therapies, those that prevent disease by addressing its origin, has been severely hindered. The peptic ulcer paradigm demonstrates that rectifying this oversight is not a philosophical exercise, but a necessary step for a full understanding of various diseases, thereby achieving genuine progress in medicine. It is the application of the CCR that makes this abstract early phase of any disease visible, revealing the true power of this universal principle. Thus, a full understanding of the pathogenesis of peptic ulcers heralds a new paradigm for medicine, one founded on a psychosomatic understanding of disease origins, paving the way for truly etiological therapies and preventive strategies that have thus far remained elusive.

### 4.5 Historical origins of the *H. pylori* misconception: how an error became prevailing consensus

Notably, as early as 1995, Rauws and Tytgat cautioned the medical community that “a strong association between infection and disease does not necessarily imply causation” (17). More than a decade later, following a thorough and detailed analysis, Hobsley and colleagues reached a similar conclusion: “*H. pylori* infection does not itself cause duodenal ulcers, but rather leads to resistance to healing” (27). In 2009, they further emphasized, with logical reasoning, that “association does not prove causation” (23). These warnings, however, have gone largely unheeded.

As demonstrated in the in-depth analysis of Section 3.2, the three lines of evidence cited to support “*H. pylori* as an etiological factor of peptic ulcers” embody clinical association and significance, but not causal relationship. Indeed, a causal relationship between *H. pylori* and peptic ulcers cannot be sustained when evaluated against Hill’s nine criteria in 1965 (50) or Wulff’s definition of etiology in 1986 (58), frameworks widely used to test the etiology of many other diseases (51–54). Remarkably, these established frameworks have never been applied in a genuinely rigorous manner to *Theory of H. pylori* before it became the prevailing consensus. In the absence of such rigorous testing, proponents of the infectious model conflated the clinical significance of *H. pylori* infection with a causal relationship between the bacterium and peptic ulcers. Despite ongoing controversy and the fact that the infectious paradigm has never explained 75 (92.6%) of the 81 key observations over the past four decades, many modern investigators have studied *H. pylori* as an etiological factor by default, accompanied by insurmountable inconsistencies. For instance, starting from the assumption of a causal role of *H. pylori* in duodenal ulcers, Ahmed and Belayneh were compelled to conclude that “other factors are also responsible for the development of duodenal ulcers” (111) The results of such studies have, in turn, been cited as the “substantial evidence supporting *H. pylori* as a major etiological factor”.

Compounding this problem is a deeply embedded epistemological habit in modern research: the tacit assumption that the most recent references are necessarily the most advanced and accurate for discovering scientific truth. The complementary principle, tracing the historical origins of a foundational theory, has rarely been employed. Consequently, findings built upon this initial conflation have been repeated thousands of times throughout the literature, creating an illusion that “substantial evidence supports *H. pylori* as an etiological factor of peptic ulcers”. The inevitable result has been four decades of stagnation in understanding the 15 major characteristics and 81 key observations of the disease, accompanied by accumulating controversies and mysteries, precisely the state we are witnessing today.

Thus, contrary to widespread perception, “substantial evidence supporting *H. pylori* as a major etiological factor” does not, in fact, exist. What exists is a vast edifice of research built upon an unrecognized category error. Indeed, over the past 40 years, peptic ulcer research has accumulated substantial evidence for the clinical significance of *H. pylori* infection, but none can be used to establish the bacterium as an etiological factor of peptic ulcers. Fortunately, guided by a new methodology originating from the CCR, the *Historical Perspective*, *Theory of Nodes* has conducted a retrospective analysis that identifies the historical origin of this long-standing conflation between clinical significance and causal relationship (32, 33). In doing so, it uncovers the limitations of *Theory of H. pylori*, rehabilitates the other four major historical etiological theories, and offers a coherent path forward for future research, making a full understanding of the pathogenesis of peptic ulcers possible.

### 4.6 Repositioning *H. pylori* within a broader etiological framework

Notably, the proposal of *Theory of Nodes* is not intended to replace the infectious paradigm, but rather to reposition it within a broader etiological framework. Within this new framework, *H. pylori* is understood not as the cause of peptic ulcers, but as a clinically significant risk factor that operates primarily in the late phase of peptic ulceration. In the absence of infection, approximately 80% of patients would likely have remained subclinical throughout their lives. However, *H. pylori* infection exacerbates symptoms, enlarges ulcers, and delays healing, thereby converting them into clinical patients. Consequently, anti-*H. pylori* therapy is highly effective in this group, markedly reducing hospitalization rates. These clinical manifestations, while demonstrating that infection and eradication have significant clinical implications, align with the mechanisms underlying the three lines of supporting evidence but do not establish causality (17). Consequently, eradication therapy remains indispensable for infected patients who show clear clinical benefit. However, *H. pylori* should no longer be studied as the primary etiological agent of peptic ulcers, as this framing has proven insufficient to explain the full scope of clinical, epidemiological, and laboratory observations, and has diverted research from more fruitful avenues for decades.

### 4.7 The comparison unmasked two systematic flaws of modern medicine

Perplexingly, despite providing critical insights, *Psychosomatic Theory* and *Stress Theory* never fully explained the pathogenesis of peptic ulcers for 70 years. After a full understanding of the pathogenesis of the disease, *Theory of Nodes* attributes this failure primarily to two unresolved issues in modern medicine. First, a viable benchmark for establishing causation has never been established. In physics, the *Universal Law of Gravitation* (by Newton in 1687) and *Mass-Energy Equation* (by Einstein in 1905) served as the definitive benchmarks, accounting for the field’s rapid advancement over the past 350 years. In contrast, however, without a benchmark in modern medicine, researchers had no foothold to judge the insights proposed by *Psychosomatic Theory* and *Stress Theory*, and attention was soon diverted by the discovery of *H. pylori*. The introduction of the Complex Causal Relationship (CCR) in 2012 posited that physics, life science, and medicine follow the same fundamental principles (31). The CCR dictates that just as all phenomena in physics are driven by invisible, intangible, and incorporeal force or energy, all diseases are driven by the invisible, intangible, and incorporeal abstract essence of the human body. Using CCR as a benchmark, only *Psychosomatic Theory* and *Stress Theory*, among all 13 historical etiologies (1), correctly identified the invisible, intangible, and incorporeal abstract essence as the cause of peptic ulcers, whereas *H. pylori* is neither invisible, intangible, nor incorporeal and thus is not the cause (32, 33). Therefore, guided by the CCR, *Theory of Nodes* pinpointed the true etiology from historical data, enabling a comprehensive understanding of the pathogenesis of peptic ulcers (**Supplementary Tables S2**–**S6**). Second, essential methodologies for guiding data analysis are still lacking. The reductionist approach, explaining a system solely through its constituent parts (112), forced the five major etiological theories to be studied in isolation, leaving them incompatible with each other. In contrast, the *Superposition Mechanism*, a novel methodology derived from the CCR, integrated these theories into a complete picture of peptic ulcer pathogenesis (36), forming the basis of *Theory of Nodes*. The application of this novel methodology has comprehensively elucidated the key observations in duodenal ulcers (32), the birth-cohort phenomenon (34) and seasonal variation (35). This consistent explanatory power establishes the *Superposition Mechanism* as an indispensable methodological complement to modern life science and medicine.

### 4.8 Broader implications: the CCR as a transformative tool for medical research

Beyond resolving the specific etiological controversies of peptic ulcers, this study demonstrates that the CCR serves dual critical roles: it acts as a robust benchmark for determining disease causation, and in doing so, it provides a powerful tool for identifying and rectifying fundamental misconceptions in medical research. This comparative analysis proposes that, while further validation is needed, *Theory of Nodes* has established itself as the most plausible etiological theory for peptic ulcers by explaining the full scope of characteristics and observations. Having done so, it is evident that the CCR enabled this breakthrough, pinpointing the true cause of peptic ulcers from the overwhelming volume of existing data (32) and unmasking the *H. pylori Misconception*. Notably, peptic ulcers served as a model disease selected to validate the CCR (32), which may now function as a universal benchmark for identifying the cause of any disease. Preliminary applications of this benchmark suggest that similar misconceptions may pervade contemporary medical research. Consequently, no disease has been fully understood, highlighting profound theoretical and methodological flaws in modern medicine. In contrast, the first application of the CCR immediately and comprehensively elucidated the pathogenesis of this model disease. This comparative effectiveness research validates the CCR and its methodologies, whose efficiency may give rise to the significant new theories and methodologies of the 21st century, driving unprecedented progress in understanding life phenomena and human diseases.

### 4.9 Limitations and future avenues for peptic ulcer research

First, the methods of systematic review were partially, but not completely, applied in this article to establish reliable benchmarks challenging the two competing theories. It is crucial to emphasize that this study is a conceptual analysis and theoretical synthesis rather than a systematic review. The six-perspective comparison directly demonstrates that this article addresses not a single, focused question but rather six, making its scope multifaceted and incompatible with the defined methodology of a systematic review, a method that synthesizes all relevant studies on a specific, focused research question. Additionally, the broad scope of this study and the fundamental heterogeneity of its search methods, employed to identify etiological theories, characteristics, and clinical observations of peptic ulcers, demonstrate that a description of article selection, together with a PRISMA flowchart (113), would not only be impractical but also fundamentally misrepresent the intellectual architecture of this work. A systematic review would, however, be the appropriate method to study each of the six perspectives in depth after the role of *H. pylori* has been redefined.

Second, this article necessarily cites numerous historical publications rather than the most recent ones. Since *Theory of H. pylori* became the prevailing model for ulcer research after 2000, earlier work on the *Psychosomatic Theory*, *Stress Theory*, *‘No acid, no ulcer’*, and *Nerve Theory* is historically situated and no longer actively pursued. *Theory of Nodes*, however, synthesizes these four foundational theories with the contemporary *H. pylori* model to achieve a full understanding of the pathogenesis of peptic ulcers. Moreover, numerous historical observations were used as benchmarks to test the validity of the two competing theories. Furthermore, to decode why the *H. pylori* model became the prevailing consensus over decades, this study examined its historical origins, specifically the three lines of supporting evidence. The explanatory power of *Theory of Nodes* demonstrates that these older sources are essential for understanding the disease’s early and intermediate phases and retain irreplaceable scientific value for comprehensively reevaluating *H. pylori*’s role in the current research landscape.

Third, among all the 15 characteristics and 81 observations elucidated by *Theory of Nodes*, only the morphology of gastric ulcers requires verification by empirical data (**Table 3**). In fact, the findings of *Stress Theory* and *Nerve Theory*, that stress-related gastric lesions are ‘brain-driven’ events, along with the well-described morphology of gastric ulcers (5, 21) and the neuroanatomy of the stomach (81), together strongly suggest the existence of such pre-existing lesions in the gastric wall. In current clinical practice, however, gastroscopy or laparoscopy can identify existing ulcers only on the mucosal or serosal surfaces of the stomach, not within the gastric wall. Moreover, research efforts have been diverted by *Theory of H. pylori* over the past 40 years, placing the findings of historical theories beyond the scope of modern ulcer research. All these constraints have limited the development of a similar pathological model to explain the morphology of gastric ulcers, leading to very slow progress in peptic ulcer research over decades. Guided by the mechanism elucidated in this study, future research can develop novel technologies to inspect these internal pathological lesions located in the interior of the gastric wall, thereby enabling the direct observation of how psychosomatic and psychological factors translate into gastric pathology in living individuals.

Fourth, as a hypothesis and theory article, this study does not aim to present new experimental or clinical data, but rather to propose a new etiological paradigm through theoretical synthesis. However, the proposed etiological theory is not speculative. It is a highly condensed summary of empirical data accumulated over the past three centuries, grounded in well-documented observations, including the 15 major characteristics and 81 key phenomena of peptic ulcers. Once its explanatory power has been widely verified, this theory can guide future empirical (clinical, epidemiological, and laboratory) research. Thus, the most urgent task at present is to establish a coherent theoretical understanding that can inform and shape new empirical investigations. Such empirical validation will, in turn, further strengthen the scientific basis of the proposed etiology. In this sense, the new theory proposed herein was born from empirical data and will ultimately return to empirical data, a necessary cycle in the advancement of science and medicine.

## 5 Conclusions

Although Marshall’s 1980s claim that ‘peptic ulcers are an infectious disease caused by *H. pylori*’ became the prevailing consensus, it coincided with 40 years of stagnant progress in the field, failing to explain 15 characteristics and 75 of 81 observations/phenomena of the disease. In contrast, *Theory of Nodes*, which integrates insights from *Psychosomatic Theory* and *Stress Theory* in 1950, addresses the full set of 15 characteristics and 81 observations/phenomena. This stark contrast suggests that the role of *H. pylori* in peptic ulcers may have been overstated in the prevailing consensus. In-depth analyses guided by *Theory of Nodes* further confirm that even in *H. pylori*-positive patients, ulceration is not initiated by the infection, supporting a non-causal role for the bacterium. Moreover, beyond the paradox presented by *H. pylori*-negative ulcers, systematic analyses reveal three critical methodological limitations in the causal proposition advanced by Marshall: interpreting clinical significance as a causal relationship, departing from Hill’s criteria for causality, and not fully adhering to Wulff’s definitions of etiology. These flaws rendered *Theory of H. pylori* ‘A Cross-century Misconception’, which has overshadowed valuable historical discoveries and diverted ulcer research for decades. Modern research on other diseases may follow similar patterns, hindering a comprehensive understanding of the pathogenesis of many diseases. Therefore, identifying such misconceptions is essential to accelerate progress in life science and medicine. Significantly, a full understanding of the pathogenesis of peptic ulcers validates the CCR and its methodologies, highlighting a psychosomatic-factor-driven early phase of any disease, a discovery that may represent a significant theoretical and methodological advance for life science and medicine in the 21st century.

## Data Availability

The dataset for this study has been published as a data article in Data in Brief, 2018 [https://doi.org/10.1016/j.dib.2018.05.022]. All relevant data are within the article and its **Supplementary Material**.
