# Supplementary material for: Reconceptualizing peptic ulcers as a psychosomatic disorder: a new etiological theory resolving a long-standing controversy surrounding Helicobacter pylori
Source: Front Psychiatry. 2026 May 15;17:1749047. doi: 10.3389/fpsyt.2026.1749047 (PMC13220400; doi:10.3389/fpsyt.2026.1749047)
Supplement: Supplementary file 1 [file DataSheet1.pdf]

Supplementary Material

Reconceptualizing peptic ulcers as a psychosomatic disorder: a new etiological theory resolving a long-standing controversy surrounding *Helicobacter pylori*

Simon Xin Min Dong; xdong084@uottawa.ca

Table of Contents

Table S1: List of 6 articles to explain 15 characteristics and 81 observations/phenomena of peptic ulcers 2

Table S2: Index of 15 elucidated characteristics of peptic ulcers 2

Table S3: Index of 6 elucidated duodenal ulcer-related observations/phenomena 4

Table S4: Index of 9 elucidated gastric ulcer-related observations/phenomena 5

Table S5: Index of 30 elucidated both gastric and duodenal ulcer-related observations/phenomena....6

Table S6: Index of 36 elucidated *H. pylori*-related observations/phenomena....10

Table S1: List of 6 articles to explain 15 characteristics and 81 observations/phenomena of peptic ulcers

Articles	Publication Information
**A1**	Dong SXM (2022). The hyperplasia and hypertrophy of gastrin and parietal cells induced by chronic stress explain the pathogenesis of duodenal ulcers. J Ment Health Clin Psychol 6: 1–12. https://doi.org/10.29245/2578-2959/2022/3.1257.
**A2**	Dong SXM (2022). A novel psychopathological model explains the pathogenesis of gastric ulcers. J Ment Health Clin Psychol 6: 13–24. https://doi.org/10.29245/2578-2959/2022/3.1259.
**A3**	Dong SXM (2022). Painting a complete picture of the pathogenesis of peptic ulcers. J Ment Health Clin Psychol 6: 32–43. https://doi.org/10.29245/2578-2959/2022/3.1261.
**A4**	Dong SXM (2025). Social and natural environmental factors cause the birth-cohort phenomenon of peptic ulcers by superposition mechanism. Int J Environ Health Res 35: 424–438. https://doi.org/10.1080/09603123.2024.2359069.
**A5**	Dong SXM (2025). Occupational and environmental factors cause the seasonal variation of peptic ulcers by Superposition Mechanism. Int J Environ Health Res 35: 2201–2215. https://doi.org/10.1080/09603123.2024.2432556.
**A6***	Dong SXM (2026). Reconceptualizing peptic ulcers as a psychosomatic disorder: a new etiological theory resolving a long-standing controversy surrounding *Helicobacter pylori.* Front. Psychiatry 17: 1749047. https://doi.org/10.3389/fpsyt.2026.1749047.

Note: 1. 'A1' refers to 'Article 1', and this abbreviation pattern applies to all other articles (e.g., A2 = Article 2, etc.). 2. In this document, '*' denotes 'this article'. 3. The eight observations/phenomena explained in this sixth article are highlighted in bold in **Tables S5** and **S6** to draw attention.

Table S2: Index of 15 elucidated characteristics of peptic ulcers

Classification	Characteristics	Explanations	Article, Page & Line No.
General (3)	1) Genetic predisposition	Individuals who are susceptible to gastric and duodenal ulcers belong to two genetically different populations. Notably, some individuals may have the genetic background for both gastric and duodenal ulcers and thus, they may have both types of ulcers simultaneously.	A2, P20, Left Column, L14-24.
	2) Etiology	Peptic ulcers are not an infectious disease caused by *H. pylori* infection, but a psychosomatic disease triggered by psychological stress.	A1, P2, Right Column, L30-48.
	3) Epidemiology	Birth-cohort phenomenon A4; Seasonal Variation A5, African enigma A6, and many others in A1, A2, A3, and A6.	A1, A2, A3, A4, A5, A6.
Clinical symptoms (6)	4) Predilection sites	The gastric antrum and lesser curvature have the most complicated functions with the largest ganglions and the greatest density of highly developed nerve plexus, determining that the local tissues at these sites receive stronger and more frequent pathogenic nerve impulses from the CNS.	A2, P17, Right Column, L17-P18, Left Column, L6.
	5) Morphology *First reported by Matthew Baillie in 1793, a 230-year-old medical mystery.*	The shape and size of submucous nodes determine the morphology of gastric ulcers. The nodes have a clear border involving the submucosa and muscular layers, determining gastric ulcers have a sharply circumscribed loss of tissue involving the mucosa, sub-mucosa, and muscular layer. Local aggressive factors in the stomach stimulate and corrode the soft tissues around the lesion, causing contraction, denaturation and necrosis. Accordingly, gastric ulcers display a characteristic 'punch out' appearance with clean edges.	A2, P17, Left Column, L47-Right Column, L30.

	6) Bleeding	If large blood vessels are close to the nodes, they may rupture due to the corrosive local aggressive factors, causing bleeding.	A2, P18, Left Column, L8-28.
	7) Perforation	A small node will not penetrate the serosa and rarely results in perforation and the symptoms are less severe. In contrast, a large node may penetrate the serosa, allowing the gastric wall to be perforated when exposed to local aggressive factors.	A2, P18, Left Column, L8-28.
	8) Relapse	Clinical patients suffer relapses of peptic ulcers because neither anti-secretory nor anti-*H. pylori* therapy is an etiological treatment and thus, the hyperplasia and hypertrophy of gastrin and parietal cells and the impact of stress have not been eliminated.	A1, P8, Right Column, L7-14.
	9) Multiplicity	The pathogenic nerve impulses from the CNS may affect the tissues at multiple locations in the stomach simultaneously due to the local nerve distribution, causing the multiplicity of submucous nodes in the gastric wall. Clinically, the disease manifests itself as the characteristic of multiplicity.	A2, P18, Right Column, L32-39.
Local aggressive factors (3)	10) Gastric acid	The hypersecretion of gastric acid is an intermediate process of duodenal ulceration, suggesting it is not a cause of duodenal ulcers, but an effect of stress triggered by personality traits or life events. Nevertheless, the hypersecretion of gastric acid is a determinant factor in the late phase of duodenal ulceration, governing many characteristics of this disease, especially the observations related to *H. pylori* and NSAIDs.	A1, P5, Right Column, L15-35.
		Gastric ulcer patients can be hypo-, normo-, or hypo-secretors of gastric acid, indicating that the hypersecretion of gastric acid is not the cause of gastric ulcers.	A2, P19, Left Column, L2-16.
	11) *H. pylori*	*H. pylori* infection is not an etiological factor/a cause of duodenal ulcer but plays a secondary role in only the late phase of duodenal ulceration, exacerbating clinical symptoms and increasing the clinical morbidity/mortality of the disease.	A1, P2, Right Column, L49-P3, Left Column, L17; A1, P7, Right Column, L34-41.
		H. pylori is not a cause but a risk factor playing a role in only the late phase of gastric ulceration, exacerbating clinical symptoms, elongating the course of ulcer, and increasing morbidity/mortality rates of gastric ulcers.	A2, P19, Left Column, L18-Right Column, L12.
	12) NSAIDs	NSAIDs usage is not an etiological factor/a cause of duodenal ulcer but plays a secondary role in only the late phase of ulceration, exacerbating clinical symptoms and increasing the clinical morbidity/mortality of the disease.	A1, P2, Right Column, L49-P3, Left Column, L17; A1, P7, Right Column, L34-41.
		NSAIDs are not an etiological factor but a risk factor playing a secondary role in only the late phase of gastric ulcers, exacerbating clinical symptoms and increasing clinical morbidity/mortality rates.	A2, P19, Right Column, L14-30.
Prognosis (3)	13) Self-healing	In most cases, especially in subclinical patients, ulcers heal up automatically due to the resolution of life events. Unfortunately, the local aggressive factors in the stomach, such as gastric acid and pepsin, *H. pylori*, NSAIDs, and mechanical abrasions may delay the healing process.	A2, P18, Left Column, L30-49.

	14) Effects of clinical treatments	The eradication of *H. pylori* would effectively cure the patients without recurrence in Cases G and I, but the eradication of *H. pylori* would not prevent the recurrence of ulcers in *H. pylori*-negative Cases D and F, or in *H. pylori*-positive Cases J and R. Cases like Cases D, F, J and R explains a dramatic decrease in ulcer recurrence after *H. pylori* eradication.	A1, P8, Right Column, L40-53.
		Medications may attenuate the impact of local aggressive factors, such as inhibiting the hyper-secretion of gastric acid, eradicating *H. pylori*, increasing the resistance of the mucosa, or protecting the injured mucosa. Thus, medications may be conducive to the regeneration and rehabilitation of local tissues, and attenuate or inhibit ulcer symptoms, resulting in resolution and less medical visits.	A2, P18, Left Column, L42-49.
	15) Hospitalization rates, morbidity, and mortality	Neither *H. pylori* infection nor NSAIDs usage is an etiological factor. Both play a secondary role in only the late phase of duodenal ulceration, exacerbating clinical symptoms and increasing clinical morbidity and mortality rates.	A1, P7, Right Column, L34-41.
		Gastric acid, *H. pylori*, and NSAIDs play a role in only the late phase of ulceration, enlarging the local lesions, exacerbating clinical symptoms, and increasing clinical morbidity/mortality rates.	A2, P18, Right Column, L42-52.

Note: 1. P6-7 = Pages 6–7; L14-24 = Lines 14–24 (this abbreviation pattern applies throughout). 2. *H. pylori* = *Helicobacter pylori*; CNS = Central Nervous System; NSAIDs = Nonsteroidal Anti-inflammatory Drugs. 3. Case G: One of 21 hypothetical cases (A–U) in *Theory of Nodes*, representing a duodenal ulcer patient without *H. pylori* infection but with gastric acid hypersecretion exceeding the ulceration threshold. For details of this case and all the others, please refer to **Figure 2** in Article 1.

Table S3: Index of 6 elucidated duodenal ulcer-related observations/phenomena

Observations/phenomena	Explanations	Article, Page & Line No.
1. 'No acid, no ulcer' (*True statement only for duodenal ulcer*).	The hypersecretion of gastric acid is the result of psychological stress and a determinant factor in the late phase of duodenal ulceration, governing multiple characteristics of this disease, especially the observations related to *H. pylori* and NSAIDS.	A1, P5, Right Column, L28-34.
2. The role of gastric acid in duodenal ulcer is further supported by the relief of pain observed after neutralization or buffering of gastric contents with alkali or food.	The late phase of duodenal ulcers is a corrosive process caused by local aggressive factors. Thus, neutralization or buffering of gastric contents with alkali or food results in the relief of pain in duodenal ulcer patients.	A1, P5, Right Column, L15-34.
3. Doll and Jones' survey suggested a positive correlation between stressful occupations and duodenal ulcer, a decreased incidence of ulcer among agricultural workers.	Psychosomatic factors or chronic stressors, such as stressful occupations, constantly provoke the neurotransmitter release in the central nervous system, resulting in the hyperplasia and hypertrophy of gastrin and parietal cells in the stomach. This potentiates individuals' response to life events. As a result, even trivialities may trigger the hypersecretion of gastric acid, leading to duodenal ulcers. In contrast, agricultural workers are less stressful in their daily life, causing a decreased incidence of duodenal ulcer.	A1, P3, Right Column, L36-P5, Left Column, L43.
4. Severe anxiety caused acid hypersecretion which, in turn, contributed to ulceration and symptoms. The fact that acid hypersecretion and symptoms abated with alleviation of stress supports this hypothesis.	The hypersecretion of gastric acid is triggered by the perception of stress via vagus nerves, and the hyperplasia and hypertrophy of gastrin and parietal cells in the early phase significantly potentiated the individual's response to life events.	A1, P5, Left Column, L37-43.

5. Rates of recurrence in patients whose initial ulcers healed during conventional anti-secretory therapy range from 60 to 100 percent per year.	Neither anti-secretory nor anti-*H. pylori* therapy is an etiological treatment and thus, the hyperplasia and hypertrophy of gastrin and parietal cells and the impact of stress have not been eliminated. The pre-existing hyperplasia and hypertrophy and the perception of stress from time to time, resulting in a high recurrence rate.	A1, P8, Right Column, L7-14.
6. Duodenal ulcer had higher incidence in large cities compared to rural areas in Africa since the 1950's.	Life in large cities is more competitive and stressful than in rural areas.	A1, P4, Left Column, L9-12.

Table S4: Index of 9 elucidated gastric ulcer-related observations/phenomena

Observations/phenomena	Explanations	Article, Page & Line No.
7. 'No acid, no ulcer' (*a misconception for gastric ulcers*).	Gastric ulceration is determined not by gastric acid, but by the formation of submucous nodes in the gastric wall. Therefore, gastric ulcer patients can be hypo-, normo-, or hyper-secretors of gastric acid.	A2, P19, Left Column, L2-16.
8. In contrast to patients with duodenal ulcer, most patients with gastric ulcer are normo-secretors or hypo-secretors. Decreased acid-peptic activity in these patients suggests impaired mucosal defence.	Gastric ulceration is determined not by gastric acid, but by the formation of submucous nodes in the gastric wall. Therefore, gastric ulcer patients can be hypo-, normo-, or hyper-secretors of gastric acid, and it is the pre-existed submucous nodes that account for the impaired mucosal defence.	A2, P19, Left Column, L2-16.
9. Gastric ulcer is a sharply circumscribed loss of tissue involving the mucosa, submucosa, and muscular layer and a characteristic "punch out" appearance with clean edges, as if it were cut by a knife.	The shape and size of submucous nodes determine the morphology of gastric ulcers. The nodes have a clear border involving the submucosa and muscular layers. As a result, gastric ulcers have a sharply circumscribed loss of tissue involving the mucosa, sub-mucosa, and muscular layer. Accordingly, gastric ulcers display a characteristic 'punch out' appearance with clean edges, as if it were cut by a knife.	A2, P17, Right Column, L19-30.
10. Gastric ulcers can be induced only in 8-30% of mouse models.	In *Theory of Nodes*, heredity determines that gastric ulcers can be induced only in 8-30% of mouse models. Genetic predisposition determines that gastric ulcer can be induced only in specially bred rat strains, reflecting the impacts of heredity on the occurrence of the disease.	A2, P20, Left Column, L14-27.
11. Gastric ulceration begin in the mucosa and extend into the wall of the stomach.	*A misconception:* gastric ulcers are not penetrating lesions beginning in the mucosa and extending into the wall of the stomach but are 'primed' by the necrotic tissues (nodes) pre-existing within the gastric wall, which make the mucosa highly susceptible to the local aggressive factors.	A2, P18, Left Column, L19-28.
12. Stress-related gastric lesions are 'brain-driven' events that may be more effectively managed through central manipulations than by altering local, gastric factors. For example, stimulation or lesions of the central nucleus of the amygdala produced or reduced gastric ulcers, respectively.	The consequence of psychological stress in gastric ulcers is the abnormal release of neurotransmitters in the CNS, leading to the transmission of pathogenic nerve impulses to the stomach.<br>Notably, in *Theory of Nodes*, what *Nerve theory* discovers is the intermediate phase of peptic ulcerations. (Please refer to Article 1)	A2, P16, Left Column, L23-Right Column, L18.
13. Development of gastric ulcers elicited by cold stress was significantly decreased by intraperitoneal injection (i.p.) pre-treatment with EDTA or α-methyl tyrosine, which depleted neurotransmitters. Gastric ulcers were significantly increased by pre-treatment with $CaCl_2$.	The consequence of psychological stress in gastric ulcers is the abnormal release of neurotransmitters in the CNS, leading to the transmission of pathogenic nerve impulses to the stomach. EDTA is a sequestering agent for removing a neurotransmitter, Ca^{2+}; α-methyl tyrosine is a competitive inhibitor for tyrosine in the production of noradrenaline and dopamine. Pre-treating animals with EDTA or α-methyl tyrosine-controlled stress-induced gastric ulcers by reducing neurotransmissions.	A2, P16, Left Column, L23-Right Column, L18.

14. The predilection sites of gastric ulcers are gastric antrum and lesser curvature.	The gastric antrum and lesser curvature have the most complicated function with the largest ganglions and the greatest density of highly developed nerve plexus. Thus, the gastric antrum and lesser curvature are the predilection sites of gastric ulcers.	A2, P17, Right Column, L41-P18, Left Column, L6.
15. Vulnerability to gastric ulceration is modulated by psychologically meaningful experiences. Repeated stress of the same type generally, but not exclusively, provides some degree of protection against ulcer during the second or later exposures.	Peptic ulcer is a psychosomatic disease triggered by psychological stress, indicating that the perception of stress determines if life events cause ulcer diseases. After repeated exposure to the same stressful life events, the individual may be mentally prepared and respond calmly without the perception of stress. Thus, psychologically meaningful experiences can modulate the vulnerability to gastric ulcers and provides some degree of protection against ulcer during the second or later exposures.	A2, P18, Right Column, L22-39.

Note: EDTA: Ethylenediaminetetraacetic acid, a versatile organic compound used primarily as a metal chelator.

Table S5: Index of 30 elucidated both gastric and duodenal ulcer-related observations/phenomena

Observations/phenomena	Explanations	Article, Page & Line No.
16. The pathogenesis of NSAID-induced peptic ulcers is complex and multi-factorial.	*A statement based on a misconception.* Since NSAIDs usage is not an etiological factor of peptic ulcers, the etiology based on NSAIDs has inevitably led to controversies and challenges in the field, and the classification of peptic ulcers based on *H. pylori* and NSAIDs does not help to elucidate the pathogenesis of peptic ulcers but make the pathogenesis of NSAIDs-induced peptic ulcers 'complex' or 'multi-factorial'.	A2, P19, Right Column, L45-51.
17. 14% of patients taking NSAIDs chronically can be found to have lesions described as gastric ulcers and 10% have lesions describes as duodenal ulcers.	In duodenal ulcer it is the hyper-secretions of gastric acid that determines if NSAIDs usage induces duodenal ulcerations, whereas in gastric ulcer it is the pre-existed submucous nodes that determine if NSAIDs usage induces gastric ulcerations. Only the NSAIDs users with the hypersecretion of gastric acid or the pre-existed submucous nodes in the gastric wall can become duodenal or gastric ulcer patients.	A2, P19, Right Column, L14-30.
18. Birth-cohort Phenomenon (*First reported by Susser and Stein in 1962*): the mortality rate of gastric ulcers in England and Wales increased at the beginning of the 20th century, reached a peak and then began to fall in the early 1950s. Similar trends for duodenal ulcers but followed ~5 years behind.	Psychological stress induced by the First World War accounts for the increasing mortality rates in the early 1910s. The high mortality rates between 1910s and 1940s were maintained by a succession of crucial social events that led to extensive psychological stress in the population. The end of the Second World War and continuous improvements in living conditions and sociopolitical environment explained the fall in the early 1950s and afterwards.	A4.
19. Once an ulcer, always an ulcer.	Clinical patients suffer relapses of peptic ulcers because neither anti-secretory nor anti-*H. pylori* therapy is an etiological treatment and thus, the hyperplasia and hypertrophy of gastrin and parietal cells and the impact of stress have not been eliminated. The pre-existing hyperplasia and hypertrophy and perception of stress from time to time, resulting in 'once an ulcer, always an ulcer'.	A1, P8, Right Column, L7-14.
20. Seasonal variation of peptic ulcer diseases (*A more-than-110-year-old mystery first reported by Berkeley Moynihan in 1910*).	The seasonal alterations periodically change multiple environmental/social factors, making a proportion of individuals in the population stressful. The psychological stress caused by each of the environmental/social factors results in its own monthly incidences and fluctuation curves. An integration of the fluctuation curves caused by all the environmental/social factors simulated the fluctuation curves in 3 typical seasonal patterns, suggesting that multiple environmental factors work together, causing the seasonal variation of peptic ulcers.	A5.

21. Patients free of ulcer distress for long periods of time were subjected to emotional trauma and feelings of insecurity during the symptom-free intervals.	For individuals with specific personality traits, even trivialities may cause severe impact, and they are more susceptible to life events. Some patients not subject to crucial life events may still suffer gastric ulceration due to personality traits that cause emotional trauma and feelings of insecurity. Long-term effects from past crucial events and specific personality traits explain the relapse of some individuals without suffering immediate crucial events.	A2, P18, Right Column, L10-15.
22. Investigations of the effects of perceived stress on physiological parameters are scarce, and the findings are often conflicting.	Questionnaire-based epidemiological surveys may have discounted many chronic stressors during peptic ulcer research, and that the essential methodology to study psychosocial factors (the abstract essence of the human body) have not been established in modern medicine.	A1, P5, Left Column, L44- P5, Right Column, L13.
23. There is no definitive study proving a causal relationship between psychological stress and the development of ulcer disease.	1. Without the Complex Causal Relationship (CCR), there is no benchmark to identify the cause of ulcer disease.	A1, P9, Left Column, L43-53.
	2. without the methodological concepts derived from the CCR, much invaluable historical data is deemed outdated and remains unanalyzed.	A1, P9, Right Column, L19-43.
	3. The birth-cohort phenomenon, seasonal variation, and African enigma are compelling evidence to support a causal relationship between psychological stress and peptic ulcer disease.	A4, A5, and A6.
24. Feldman's multidimensional case-controlled study found that ulcer patients exhibited significantly more emotional distress in the form of depression and anxiety. Hypochondriasis, a negative perception of their life events, dependency, and lowered self-confidence were the four variables that best discriminated ulcer patients from controls.	Peptic ulcers are a psychosomatic disease triggered by psychological stress. Psychosomatic factors made individuals susceptible to psychological stress caused by current life events, leading to peptic ulceration.	A1, P2, Right Column, L30-48.
25. Peptic ulcers are a rare disease in childhood.	Most children do not comprehend the gravity of life events or how the events affect them in the future. Major life events and psychological distress during childhood and adolescence might differ in some fundamental ways from adulthood. Children are comfortable in their own skin and not as effected by social expectations. Their different feelings and mindset, as compared to adults suggest they experience less stress, resulting in the rarity of peptic ulcers in children.	A3, P35, Left Column, L1-13.
26. Although gastric ulcer and duodenal ulcer share something in common, they are believed to be different diseases.	Table 1 in Article 2 listed all the similarities and differences between the two diseases. Gastric and duodenal ulcer patients belong to two genetically different populations. Both are psychosomatic diseases triggered by psychological stress with abnormal neurotransmitters in the CNS, resulting in pathogenic nerve impulses to the stomach. Stomach is the effector organ for both diseases, but the outcomes differ: duodenal ulcers result from gastric acid hypersecretion, whereas gastric ulcers arise from the formation of submucosal nodes in the gastric wall. *H. pylori* and NSAIDs play secondary roles in only the late phase and are not essential for both diseases. Acute stress directly causes gastric ulcers, whereas chronic stress requires ~5 years to induce the hyperplasia and hypertrophy of gastrin and parietal cells in the stomach for duodenal ulcers.	A2, P20, Table 1.
27. The final stage of ulceration is a corrosive rather than an infectious process.	The late phase of duodenal ulcers is a corrosive process caused by local aggressive factors, including gastric acid and pepsin, *H. pylori*, mechanical abrasion, and chemical erosion from medications such as NSAIDs.	A1, P5, Right Column, L15-34.
	The late phase of gastric ulceration is also a corrosive process caused by local aggressive factors in the stomach, such as gastric acid and pepsin, *H. pylori*, mechanical abrasion, NSAIDs and other medications.	A2, P17, Left Column, L47-L51.

28. The gastric acid secretion of duodenal ulcer patients is much higher than a normal person, but only 7–8.5% of the duodenal ulcer patients are suffering from gastric ulcer simultaneously.	Gastric ulceration is determined not by gastric acid, but by the formation of submucous nodes in the gastric wall. Therefore, gastric ulcer patients can be hypo-, normo-, or hyper-secretors of gastric acid. Some individuals may have the genetic background for both gastric and duodenal ulcers and thus, they may have both types of ulcers simultaneously.	A2, P19, Left Column, L2-16.
29. Severe emotional stress may contribute to ulcer perforation and bleeding in some patients.	The degree to which individuals suffer negative impacts from life events determined the size and depth of the nodes. A moderate stress causes small and superficial ulcers, but severe emotional stress may cause ulcer perforation and bleeding.	A2, P18, Left Column, L19-28.
30. Many uncomplicated lesions heal in spite of the presence of acid gastric content, as shown by the "spontaneous" remissions of the disease and by the healed scars found at x-ray and at autopsy; however, the healing of peptic ulcer is much more rapid when the lesion is protected from the action of acid gastric juice.	The impacts of the pathogenic nerve impulses are relieved at the ulcerated location once the distal ends of the nerves necrotize. As a result, gastric ulcers may heal up automatically via the regeneration of local tissues without treatment. Medications may attenuate the impact of local aggressive factors, such as inhibiting the hyper-secretion of gastric acid, eradicating *H. pylori*, increasing the resistance of the mucosa, or protecting the injured mucosa. Thus, medications may be conducive to the regeneration and rehabilitation of local tissues, and attenuate or inhibit ulcer symptoms, resulting in resolution and less medical visits.	A2, P18, Left Column, L30-49.
31. Autopsy reports showed: 20-29% of males and 11-18% of females were found to have suffered from ulcers in the past or present.	Heredity is an innate factor playing a baseline role in peptic ulcers, which predisposes a proportion of individuals in the population to an ulcer personality. Thus, not all the individuals suffer peptic ulcers.	A3, P37, Right Column, L12-27.
	From the 1900's to the 1950's, men were often the direct participants of various social conflicts such as wars and financial crisis, and they were more likely to bear economical pressures than women, explains a higher percentage of males (20-29%) have suffered from ulcers in the past or at present than females (11-18%) in autopsy reports56–58.	A3, P37, Right Column, L41-52.
32. It is believed that, not only should the prognosis and assessment of ulcer have mental assessment, but the treatment without mind adjustment is also incomplete.	A complete cure of peptic ulcers depends on the resolution of life events and/or the resolution of mental health concerns;	A3, P38, Right Column, L2-7.
	Promoting social harmony and mind adjustment are essential etiological therapy for a complete cure of peptic ulcers without relapse.	A3, P39, Left Column, L35-43.
33. Peptic ulcer patients may have "ulcer personality", such as immaturity, impulsivity, and feelings of social isolation and alienation.	Heredity is an innate factor playing a baseline role in peptic ulcers, which predisposes a proportion of individuals in the population to an ulcer personality.	A3, P37, Right Column, L12-27.
**34. In a relatively isolated group of Australian aboriginals, peptic ulcers were rare. In a 2-year study of Pima Indians in North America, no peptic ulcer was found.**	**The low incidence of peptic ulcers in indigenous groups was due to a pastoral lifestyle with fewer social conflicts and therefore, they were leading a less stressful life.**	**A6, Section 3.3. P5 Right Column L50 - P6 Left Column L1.**
**35. In contrast to Pima Indians, 10% of Caucasians develop peptic ulcers.**	**The higher incidence in Caucasians resulted from modern societal pressures like financial crises, unemployment, or competition.**	**A6, Section 3.3. P6, Left Column, L2-4.**
36. To date, no consistent pattern of factors, in either host or organism, has been identified that successfully predicts which infected persons will subsequently have ulcer disease.	According to the complete picture painted in Article 3, the occurrence of peptic ulcers is predictable if the 4 preconditions are satisfied: heredity; Hyperplasia and hypertrophy in the stomach and/or a negative life-view, psychological stress, *H. pylori* infection and NSAIDs usage	A3, P37, Right Column, L1-P38, Left Column, L41.
37. The relationship between life events stress, psychological factors and peptic ulcer diseases is not clearly established at the present time and warrants further study.	Without the CCR, there is no benchmark to identify the cause of disease. As a result, no disease has been fully understood.	A1, P9, Left Column, L43-53.
	Without the CCR, the methodologies essential for data analysis have never been established, resulting in many unsolved mysteries and massive challenges.	A1, P9, Right Column, L19-43.

38. Richard emphasized the different etiology of gastric and duodenal ulcers and persons with gastric and duodenal ulcers differ epidemiologically, behaviourally, and genetically.	Table 1 listed the similarities and differences between duodenal and gastric ulcers.	A2, P20, Table 1.
39. Gastric ulcer was more frequent than duodenal ulcer, 4G:1D in 1900 versus 10D:1G currently. More women than men had the disease, but now it has become reversed; from 3F:1M to 4M:1F for gastric ulcer and 10M:1F for duodenal ulcer.	These reversions are due to the profound changes in social environments during the past century. The civilization and urbanization, etc. result in the reversion of gastric: duodenal ratio. Before 1900, the life threats or survival-related challenges and the severe shortages of supplies determined that the majority of peptic ulcer cases were gastric ulcer patients. However, after 1900, especially after World War II, most of the threats to survival disappeared and the steady growth of people's living standard lead to the decreased incidence of gastric ulcers, whereas the fast rhythm of modern life, fierce social competition, and unhealthy lifestyle, etc., primarily induce duodenal ulcers. The reversion of the sex ratio can be explained by the liberation and improved social status for women.	A3, P34, Left Column, L33-Right Column, L11.
40. Stress ulcers in the rat are primarily gastric rather than duodenal, the latter typically requiring additional artificial chemical potentiation (e.g., histamine).	It may take ~5 years to induce the hyperplasia and hypertrophy of gastrin and parietal cells in the stomach. As a result, it is hard to duplicate this chronic pathophysiological process in the lab, making it difficult to establish animal models for stress-induced duodenal ulcers.	A1, P5, Left Column, L14-20.
	Duodenal ulcer is a chronic disease associated with individual's daily behaviors, and multiple psycho-neuro-endocrine organs were involved. Thus, stress-induced duodenal ulcer is hardly reproduced in animal models due to short lifespans.	A1, P9, Left Column, L15-20.
	The psychological stress triggering gastric ulcers is usually short-term and acute. This kind of stress can be simulated in labs and therefore, stress-induced ulcers in labs are usually gastric ulcers.	A2, P16, Left Column, L2-11.
41. Many ulcer patients and some physicians believe that symptomatic exacerbations of peptic ulcer disease occur during or shortly after stressful events.	Peptic ulcers are a psychosomatic disease triggered by psychological stress. Psychosomatic factors made individuals susceptible to psychological stress caused by current life events, leading to peptic ulceration.	A1, P2, Right Column, L30-48.
42. Mental disorders (or stress) are associated with increased rates of peptic ulcer diseases.	Peptic ulcers are a psychosomatic disease triggered by psychological stress. Psychosomatic factors, which focuses more on the impact of mental health related to past life experience, made individuals susceptible to psychological stress caused by current life events, leading to peptic ulceration.	A1, P2, Right Column, L30-48.
43. The spontaneous remissions and relapses of peptic ulcers have never been explained.	Clinical patients suffer the relapse of peptic ulcers, because neither anti-secretory nor anti-*H. pylori* therapy is an etiological treatment and thus, the impact of stress caused by personality traits or negative life events has not been eliminated.	A1, P8, Right Column, L7-14.
	Chronic stress-induced hyperplasia and hypertrophy of gastrin and parietal cells are the crux of duodenal ulcer issue, determine the spontaneous remissions and relapse of duodenal ulcer, but this psychopathological mechanism has never been elucidated in modern medicine. *The etiology of peptic ulcer has never been identified, causing the problem.*	A1, P9, Left Column, L15-26.
44. The pathophysiology of peptic ulcer has centred on an imbalance between aggressive and protective factors.	The mechanisms of diabetes and cardiomegaly suggest that the origins of disease and the pathological changes could present in different organs. Therefore, the traditional etiological concept (The pathophysiology of peptic ulcer has centred on an imbalance between aggressive and protective factors) is not sufficient to discover the cause of disease, and peptic ulcers may not be a local disease, but a general disease due to abnormalities in other parts of the human body.	A3, P33, Right Column, L4-24.

45. No single theory in history could fully explain the pathogenesis of peptic ulcers.	Each of the single theory focused on only one area of the human body, and there is no holistic review of peptic ulcers before.	A1, P3, Left Column, L20-29.
	Without the CCR, there is no benchmark to identify the true cause of disease. In addition, without the CCR, the methodologies for data analysis have never been established.	A1, P9, Left Column, L43-53.
	Although each of the historical theory did make important discoveries and proved useful to understand peptic ulcers, they were overshadowed by the discovery of *H. pylori*.	A1, P9, Right Column, L19-43.

Note: CCR = Complex Causal Relationship - A universal law proposed by the author (2012) in a book entitled ***Philosophical Principles of Life Science*** for identifying causes of life phenomena and human diseases. (See Article 1.)

Table S6: Index of 36 elucidated *H. pylori*-related observations/phenomena

Observations/phenomena	Explanations	Article, Page & Line No.
**46. African Enigma: The *H. pylori* infection rate is high (close to 100%) throughout Africa, but the prevalence of duodenal ulcer varied in different parts of the continent**	**The regional differences in morbidity rates across Africa stem not from *H. pylori*, but from varied and stressful environmental factors, such as political stability, economic development, and social welfare disparities. The default assumption that '*H. pylori* is the cause of peptic ulcers' gave rise to the African enigma.**	**A6, Section 3.5.1. P7, Left Column, L33-38;**
47. Only the presence of duodenal ulcers, and not gastric ulcers, was associated with increasing *H. pylori* density. The association between gastric ulcers and *H. pylori* infection is less clear.	Since each of the 3 local aggressive factors partially contributes to the corrosive intensity, and duodenal ulceration is caused by the total corrosive intensity, the higher the density of *H. pylori*, the higher the total corrosive intensity. As a result, the infection of *H. pylori* exacerbates clinical symptoms and increases the clinical morbidity/mortality of the disease. Cases B to J explain why *H. pylori* density is associated with the morbidity rate of duodenal ulcers.	A1, P7, Right Column, L34-48.
	In gastric ulcers it is not the total corrosive intensity of local aggressive factors, but the formation of submucous nodes that determines the occurrence of gastric ulcers. Therefore, *H. pylori* has a dose-effect on only duodenal ulcers, whereas gastric ulcers' association with *H. pylori* is less clear than duodenal ulcers.	A2, P19, Right Column, L3-12.
**48. Only 27% of symptomatic children with peptic ulcers were *H. pylori* positive.**	**Younger patients, with fewer cumulative life events due to short lifetimes, are less likely to develop ulcers. Children with hyperplasia and hypertrophy of gastrin and parietal cells or a negative life-view are rare, explaining the low incidence in this age group.**	**A6, Section 3.5.2. P7, Right Column, L19-23.**
**49. 48% of patients developed ulcers within six months of healing, but the re-infection rate after eradication was very low (<2%).**	**If *H. pylori* were the cause, its eradication would prevent all relapses. The consistent ~20% relapse rate post-eradication, which vastly exceeds the long-term reinfection rates as low as ~1% per year, indicates that relapse cannot be attributed solely to reinfection but implicates 'other factors'. *Theory of Nodes* proposes that *H. pylori* plays a secondary role predominantly in the late phase of ulceration. Consequently, eradication therapy does not address the primary triggers, namely, the preexisting hyperplasia and hypertrophy of gastrin and parietal cells or the underlying negative life-view. These primary factors, which precisely constitute the 'other factors', can reactivate acid hypersecretion and node formation, leading to relapse.**	**A6, Section 3.2.3. P5, Right Column, L5-18.**
50. In developing countries with uniformly high prevalence of *H. pylori* infection, there are marked regional differences in the prevalence of duodenal ulcers, which could not be explained by the more toxic CagA and VacA *H. pylori* strains.	*H. pylori* infection is not the cause of duodenal ulcers but plays a secondary role in only the late phase of ulceration and thus, *H. pylori* strains such as CagA, VacA, and SecA cannot explain the higher prevalence of duodenal ulcers.	A1, P8, Left Column, L2-16.

51. In the countries with low prevalence of *H. pylori*, 30-40% or more of duodenal ulcer patients are *H. pylori*-negative, and the absence of *H. pylori* infection in early cases of duodenal ulcers was also reported.	*H. pylori* infection is not the cause of duodenal ulcers but plays a secondary role in only the late phase of ulceration.	A1, P8, Left Column, L2-16;
	Cases D, F, L, and N support the finding that in some countries, 30-40% of duodenal ulcer patients were *H. pylori*-negative and up to 35% of 387 infected patients failed to respond to standard anti-*H. pylori* therapy.	A1, P8, Right Column, L19-27.
52. The role of *H. pylori* in peptic ulcers is controversial.	Without the CCR, there is no benchmark to identify the cause of disease, and the methodologies essential for data analysis have never been established, resulting in many unsolved mysteries and massive challenges.	A1, P9, Left Column, L43-53.
	After the application of the CCR in *Theory of Nodes*, the role of *H. pylori* in peptic ulcers has been elucidated and therefore are no longer controversial.	A1, P9, Right Column, L19-43.
53. '*H. pylori* is the most important aetiological factor so far described for duodenal ulcer'.	*This is a misconception.* The hyper-secretion of gastric acid is a determinant factor in the late phase of duodenal ulceration; Administering cysteamine or propionitrile, which effectively stimulates the hypersecretion of gastric acid in rats, can induce acute and chronic duodenal ulcers in normal rats, whereas the inoculation of *H. pylori* alone cannot, suggesting it is not *H. pylori* but gastric acid that plays a primary/decisive role in the pathogenesis of duodenal ulcers.	A1, P5, Right Column, L36-52.
54. How *H. pylori* infection can lead to ulceration is unknown.	*This statement is based on a misconception.* Without the hypersecretion of gastric acid, the corrosive intensity caused by either *H. pylori* or NSAIDs could not reach the threshold of ulceration. *H. pylori* alone cannot lead to ulceration and thus, the etiology based on *H. pylori* infection would never be able to explain peptic ulcerations.	A1, P7, Right Column, L44-53.
55. No *H. pylori*, No Ulcer; peptic ulcer is an infectious disease caused by *H. pylori*.	*This is a misconception.* Neither *H. pylori* infection nor NSAIDs usage is essential for ulceration. Therefore, duodenal ulcers may occur in the absence of *H. pylori* and/or NSAIDs, leading to idiopathic (*H. pylori*-negative and non-NSAIDs user) ulcer patients.	A1, P5, Right Column, L38-48.
56. In spite of a high prevalence of *H. pylori* infection worldwide, the incidence of duodenal ulcer disease in both adults and children is low in comparison.	Case B is applicable to most *H. pylori* infected individuals, but only those also impacted by the hyper-secretions of gastric acid due to psychological stress (Case G) will have the chance to become ulcer patients.	A1, P8, Left Column, L17-23.
57. Kato and colleagues' retrospective analysis found that *H. pylori* prevalence in gastric ulcer did not reach 50%; they concluded while *H. pylori* infection appears to be a risk factor in gastric ulcer, other causes are responsible for most cases. Only 56-96% of gastric ulcer patients are *H. pylori*-positive, so other factors must be involved.	The formation of submucous nodes is irrelevant to *H. pylori* infection but determined by only the pathogenic nerve impulses from the CNS. Without infection, gastric ulcers may occur if the individual is severely impacted by psychological stress. Therefore, more than 50% of clinical gastric ulcer patients can be *H. pylori* negative. The pre-existed pathological lesions in the gastric wall due to psychological stress account for the other unidentified factors. On the other hand, because gastric ulceration is determined not by *H. pylori* infection, but by the formation of submucous nodes induced by psychological stress, even though the infection rate in the population is as high as 90%, only the individuals affected by psychological stress become ulcer patients, leading to the low incidence of gastric ulcers despite a high prevalence of *H. pylori* infection.	A2, P19, Left Column, L32-49.
58. Despite the fall in prevalence of *H. pylori* infection, the attributable risk of *H. pylori* infection in peptic ulcer disease has not changed.	Since *H. pylori* infection is not the cause of peptic ulcers, the attempt to explain an epidemiological observation by *H. pylori* infection would inevitably result in the inconsistency.	A2, P19, Left Column, L49-Right Column, L2.

59. There was a significantly higher incidence of idiopathic duodenal ulcers in the younger generation.	The younger the individuals, the less chances they get infected with *H. pylori* and/or become a NSAIDs users, resulting in the significantly higher incidence of idiopathic duodenal ulcers.	A2, P19, Right Column, L51-P20, Left Column, L3.
60. There are basically three different types of peptic ulcer: *H. pylori*-related peptic ulcer; NSAID-related peptic ulcer; and non-*H. pylori*, non-NSAID ulcer.	*This classification is because the cause of peptic ulcers has never been identified in modern medicine.* It overlooks the primary/decisive factor in the occurrence of duodenal ulcers, the hypersecretion of gastric acid. *The causal roles of psychosomatic factors and psychological stress were also neglected.*	A1, P8, Right Column, L14-18.
**61. A relatively isolated group of Australian aboriginals have virtually no *H. pylori* infection and hardly any peptic ulcer disease.**	**The low incidence of peptic ulcers in indigenous groups was due to a pastoral lifestyle with fewer social conflicts and therefore, they were leading a less stressful life.**	**A6, Section 3.3. P5 Right Column L50 - P6 Left Column L1.**
62. Up to 20% of ulcer patients suffer a relapse despite successful eradication of their infections, suggesting that *H. pylori* was not the cause of their original ulcers.	Cases D and F were caused simply by the hypersecretion of gastric acid due to psychological stress, which explain 20-40% of patients with idiopathic ulcers (*H. pylori*-negative and non-NSAIDs user) and the eradication of *H. pylori* can not prevent the relapse of these cases.	A1, P8, Left Column, L42-46.
63. Difference in virulence of *H. pylori* strains (cag- and cag+) has been considered a putative explanation to why only a minority of infected population develop peptic ulcers.	*H. pylori* infection is not the cause of duodenal ulcers but plays a secondary role in only the late phase of ulceration and thus, *H. pylori* strains such as CagA, VacA, and SecA, cannot explain the higher prevalence of duodenal ulcers.	A1, P8, Left Column, L2-16.
64. *H. pylori* infection in rats was successful and was accompanied by a mild to moderate mucosal inflammation. After *H. pylori* inoculation, an ulcer was induced in the oxyntic mucosa of both infected and uninfected rats by exposing the serosal side to acetic acid.	For those individuals with severe hyperplasia and hypertrophy of gastrin and parietal cells, the hypersecretion of gastric acid alone is sufficient to induce duodenal ulcerations. In that case, neither *H. pylori* infection nor NSAIDs usage is essential for ulceration. Therefore, duodenal ulcers may occur in the absence of *H. pylori* and/or NSAIDs, leading to idiopathic (*H. pylori*-negative and non-NSAIDs user) ulcer patients. Administering cysteamine or propionitrile, which effectively stimulates the hypersecretion of gastric acid in rats, can induce acute and chronic duodenal ulcers in normal rats, whereas the inoculation of *H. pylori* alone cannot, further indicating that gastric acid plays a more important role than *H. pylori* in duodenal ulcerations.	A1, P5, Right Column, L36-52.
**65. More than 95% patients with duodenal ulcers and more than 80% patients with gastric ulcers are infected with *H. pylori*.**	***H. pylori* is not a cause but a risk factor that plays a secondary role only in the late phase of peptic ulceration, exacerbating ulcer symptoms, delaying healing, and increasing morbidity and mortality rates. Consequently, infected individuals are more likely to seek medical care and become clinical patients due to exacerbated symptoms, while uninfected individuals often remains subclinical with milder symptoms. This skews epidemiological surveys: many *H. pylori*-negative subclinical patients are excluded from the calculation, inflating infection rates among clinical patients (Table 1).**	**A6, Section 3.2.1. P5, Left Column, L3-12.**
66. The corresponding ulcer areas in the *H. pylori*-infected rats were significantly larger in the infected than in the uninfected rats, and ulcer healing was delayed in the infected rats. Eliminating *H. pylori* accelerates the healing of ulcer.	Only after the submucous nodes have come into being, could *H. pylori* have the chance to corrode the tissues at the site, enlarging the lesions and delaying the regeneration and rehabilitation of local tissues.	A2, P19, Left Column, L18-31.
	Eradicating *H. pylori* attenuates the impact of local aggressive factors, is conducive to the regeneration and rehabilitation of local tissues, and mitigates or inhibits ulcer symptoms, resulting in resolution and less medical visits.	A2, P18, Left Column, L42-49.

67. Eradication of *H. pylori* in gastric ulcer patients has also been shown to be associated with a significant reduction in ulcer relapse rate, compared with those who remain infected.	Medications, such as proton pump inhibitors for gastric acid reduction and antibiotics for *H. pylori* eradication, may mitigate symptoms, reduce the morbidity rate, and delay relapse to a certain degree, and many cases were diagnosed as healed, but the real cause of gastric ulcers, psychosocial stress, was not removed. As a result, relapse may occur during treatments.	A2, P18, Right Column, L3-10.
68. Clinical data reported that the recurrence rate is as high as 74-80% in *H. pylori* positive group of duodenal ulcer patients who have healed, but the negative group is only 0-28%. The discrimination was remarkable.	The eradication of *H. pylori* would effectively cure the patients without recurrence in Cases G and I, which are representative of most clinical patients. However, the eradication of *H. pylori* would not prevent the recurrence of ulcers in *H. pylori*-negative Cases D and F, or in the *H. pylori*-positive Cases J and R, where the damage caused by the bacterium was not a determinant factor in ulceration. It is more common to identify cases similar to Cases G and I in clinical patients, whereas cases like Cases D, F, J and R occur less frequently, which explains the dramatic decrease of ulcer recurrences after *H. pylori* eradication.	A1, P8, Right Column, L40-53.
69. A negative interaction between *H. pylori* and NSAIDs on duodenal ulcers suggests that *H. pylori* reduces the development of ulcers in NSAIDs users.	*H. pylori* upregulates the expression of COX-2 and subsequently results in the biosynthesis of gastroprotective prostaglandin, which in turn offsets the corrosive intensity of NSAIDs and thus is conducive to the healing of NSAIDs-induced ulcers, causing '*H. pylori* reduces the development of ulcers in NSAIDs users'.	A1, P7, Left Column, L46-52.
70. ~20% of peptic ulcers in the Polish population are unrelated to *H. pylori* and NSAIDs use (idiopathic ulcers).	For individuals with severe hyperplasia and hypertrophy of gastrin and parietal cells, the hypersecretion of gastric acid alone is sufficient to induce duodenal ulcerations. In that case, neither *H. pylori* infection nor NSAIDs usage is essential for ulceration. Therefore, duodenal ulcers may occur in the absence of *H. pylori* and/or NSAIDs, leading to idiopathic (*H. pylori*-negative and non-NSAIDs user) ulcer patients.	A1, P5, Right Column, L36-52.
71. The prevalence of *H. pylori* in patients with bleeding ulcers may be 15~20% lower than in patients with non-bleeding ulcers.	Bleeding indicates patients are more likely to be seriously impacted by psychological stress with severe hyperplasia and hypertrophy of parietal and gastrin cells as Cases F and *H. pylori* infection is not essential for ulceration. In contrast, non-bleeding ulcer suggests the patients are more likely to be slightly impacted by stress with mild hyperplasia and hypertrophy, and *H. pylori* infection is essential to increase the total corrosive intensity for the induction of ulcers as Case G. As a result, clinical bleeding ulcer patients are more likely to be Case F without *H. pylori* and non-bleeding patients are more likely to be Case G with *H. pylori* infection.	A1, P8, Left Column, L47- Right Column, L7.
72. The eradication of *H. pylori* reduces the rate of re-bleeding in patients with ulcer disease.	The eradication of *H. pylori* decreases the total corrosive intensity, causing mitigated symptoms, lowered morbidity and the rates of re-bleeding in ulcer patients.	A1, P7, Right Column, L41-44.
73. How *H. pylori* infection affects gastric acid secretion is still unclear.	The hypersecretion of gastric acid is unrelated to any of the local aggressive factors, such as *H. pylori* infection, NSAIDs and other medications, but potentiated by the hyperplasia and hypertrophy of one or more endocrine organs and triggered by psychological stress due to personality traits or life events.	A1, P5, Right Column, L20-35.
74. The incidence of peptic ulcers was higher in *H. pylori*-infected patients than in the *H. pylori*-negative group.	Since each of the 3 local aggressive factors partially contributes to the corrosive intensity, and duodenal ulceration is caused by the total corrosive intensity, the higher the density of *H. pylori*, the higher total corrosive intensity. As a result, the infection of *H. pylori* exacerbates clinical symptoms and increases the clinical morbidity/mortality of the disease.	A1, P7, Right Column, L34-41.

75. Duodenal acid load determines whether *H. pylori* can cause duodenal ulcer.	The hypersecretion of gastric acid is the determinant factor in the late phase of duodenal ulceration, governing multiple characteristics of this disease, especially the observations related to *H. pylori* and NSAIDs.	A1, P5, Right Column, L25-35.
76. The increase in *H. pylori* density is related to the presence of duodenal ulcer disease.	Since each of the 3 local aggressive factors partially contributes to the corrosive intensity, and duodenal ulceration is caused by the total corrosive intensity, the higher the density of *H. pylori*, the higher total corrosive intensity. As a result, the infection of *H. pylori* exacerbates clinical symptoms and increases the clinical morbidity/mortality of the disease.	A1, P7, Right Column, L34-41.
77. *H. pylori*-negative duodenal ulcers were associated with a poorer prognosis mainly because of a higher rate of ulcer and symptom relapse.	To reach the ulceration threshold without the corrosive intensity from *H. pylori*, the hypersecretion of gastric acid needs to be higher in *H. pylori*-negative patients. The higher hypersecretion in Cases D and F indicates more severe negative impacts from psychosocial stress than in Cases G and I. Additionally, the damage from *H. pylori* can be relieved by antibiotics, as in Cases G and I, decreasing the corrosive intensity in these patients, whereas Cases D and F cannot achieve the same therapeutic effect. Therefore, there is a poorer prognosis and a higher relapse rate in *H. pylori*-negative patients.	A1, P8, Left Column, L31-41.
78. When *H. pylori* persisted, 61% of duodenal ulcers healed and 84% relapsed. When *H. pylori* was cleared 92% of ulcers healed and only 21% relapsed during the 12 months follow-up period.	Since each of the 3 local aggressive factors partially contributes to the corrosive intensity and duodenal ulceration is caused by the total corrosive intensity, the higher density of *H. pylori*, the higher total corrosive intensity. As a result, *H. pylori* infection exacerbates clinical symptoms and increases the clinical morbidity/mortality of the disease. In contrast, the eradication of *H. pylori* decreases the total corrosive intensity, causing mitigated symptoms, lowered morbidity and the rates of re-bleeding in ulcer patients.	A1, P7, Right Column, L34-44.
79. Jyotheeswaran and colleagues from greater Rochester, New York, reported a 48% prevalence of *H. pylori*-negative duodenal ulcers in white patients and 15% in non-white patients, with an overall negative prevalence of 39%. Parsonnet's meta-analysis indicates the overall prevalence of *H. pylori*-negative duodenal ulcers is 40%.	For those individuals with severe hyperplasia and hypertrophy of gastrin and parietal cells, the hypersecretion of gastric acid alone is sufficient to induce duodenal ulcerations. In that case, neither *H. pylori* infection nor NSAIDs usage is essential for ulceration. Therefore, duodenal ulcers may occur in the absence of *H. pylori* and/or NSAIDs, leading to idiopathic (*H. pylori*-negative and non-NSAIDs user) ulcer patients. *The prevalence of H. pylori-negative ulcer patients varies regionally, and it is very likes that some regions have higher percentages of H. pylori-negative patients.*	A1, P5, Right Column, L36-52.
80. A review by van der Voort and colleagues suggests that existing data are consistent with a causal role for *H. pylori* in stress ulcer formation.	*A statement originated from a misconception. 'A causal role for H. pylori in stress ulcer' indicates that without stress, H. pylori alone can not induce ulcer.* Administering cysteamine or propionitrile, which effectively stimulates the hypersecretion of gastric acid in rats, can induce acute and chronic duodenal ulcers in normal rats, whereas the inoculation of *H. pylori* alone cannot, further indicating it is not *H. pylori* but gastric acid that plays a primary/decisive role in the pathogenesis of duodenal ulcer.	A1, P5, Right Column, L43-52.
	Case B in Article 1 is applicable to most *H. pylori* infected individuals, but only those also impacted by the hyper-secretions of gastric acid due to psychological stress (Case G) have the chance to become ulcer patients. *That explained why the statement "a causal role for H. pylori in stress ulcer formation" is questionable.*	A1, P8, Left Column, L17-25.
**81. In 1984, Dr. Barry Marshall drank a concoction made from cultured *H. pylori* and came down with gastritis that could be cured with antibiotics.**	**He expected to develop an ulcer, potentially years later, but instead only experienced gastritis, which was later cured with antibiotics. This outcome, gastritis without ulcer formation, is inconsistent with the prediction that *H. pylori* is the primary cause of peptic ulcers.**	**A6, Section 3.6.6. P9, Left Column, L12-18.**
